# Isolation and Identification of Secondary Metabolites in *Rheum tataricum* L.fil. Growing in Kazakhstan and Surveying of Its Anticancer Potential

**DOI:** 10.3390/molecules30142978

**Published:** 2025-07-15

**Authors:** Aiman A. Turgunbayeva, Nurgul A. Sultanova, Mohammad Saleh Hamad, Victor A. Savelyev, Elena I. Chernyak, Irina Yu. Bagryanskaya, Mikhail A. Pokrovsky, Andrey G. Pokrovsky, Nadezhda G. Gemejiyeva, Elvira E. Shults

**Affiliations:** 1 Faculty of Natural Sciences, Department of Chemistry, L.N. Gumolyov Eurasian National University, Satpayeva Str., 2, 010008 Astana, Kazakhstan; nureu@mail.ru; 2 Zelman Institute for the Medicine and Psychology, Novosibirsk State University, Pirogova Str., 1, 630090 Novosibirsk, Russia; m.khamad@g.nsu.ru (M.S.H.); agpok@inbox.ru (A.G.P.); 3 Novosibirsk Institute of Organic Chemistry, Siberian Branch of the Russian Academy of Sciences, 630090 Novosibirsk, Russia; vicsav@nioch.nsc.ru (V.A.S.); chernyak@nioch.nsc.ru (E.I.C.); bagryan@nioch.nsc.ru (I.Y.B.); miha.pokrovsky@gmail.com (M.A.P.); 4 Institute of Botany and Phytointroduction FWC of the Ministry of Ecology and Natural Resources of the Republic of Kazakhstan, Timiryazev Str., 36 D, 050040 Almaty, Kazakhstan; ngemed58@mail.ru

**Keywords:** *Rheum tataricum*, phenylbutanoids, hydroxylated phenylpropanoids, stilbenes, X-ray analyses, cytotoxicity

## Abstract

*Rheum tataricum* L.fil., known for its high tolerance to drought, salinity, and nutritional deficiency, is the least studied species of wild rhubarb. Extract of roots and rhizomes of *R. tataricum* has been traditionally used for the treatment of different diseases such as liver, kidney, womb, and bladder diseases and also relapsing fever. An ethanol extract of the roots of *R. tataricum* was prepared and further successively fractionated by extraction with *tert*-butyl methyl ether (*TBME*) and ethyl acetate (*EtOAc*). The obtained extract fractions were subjected to a series of chromatographic separations on silica gel for the isolation of its individual compounds. A total of 12 individual compounds, 2-*O*-β-*D*-glucopyranoside of *R*-(4-hydroxyphenyl)-2-butanol (rhododendrin) **1**, gallic acid **2**, 2-*O*-β-*D*-glucopyranoside of *S*-4-(4-hydroxyphenyl)-2-butanol (*epi*-rhododendrin) **3**, their aglycones (-)-(2*R*)-rhododendrol **4** and (+)-(2*S*)-rhododendrol **5**, gallotannin β-glucogallin **6**, chlorogenic acids (3,5-di-*O*-caffeoylquinic acid **7** and 5-*O*-caffeoyl-3-*O*-(*p*-coumaroyl) quinic acid **8**), 4-(4-hydroxyphenyl)-2-butanon (raspberry ketone) **9** and three stilbenes (rhaponticin **10**, desoxyrhaponticin **11** and resveratroloside **12**), were isolated and characterized. The structure of desoxyrhaponticin **11** was confirmed by X-ray diffraction analyses. The results of in vitro biological assays (the MTT test) showed that ethanol extract *Rheum tataricum* was non-toxic against the normal epithelial VERO cells. The isolated compounds **1**, **4**, **11** and **12** exhibited cytotoxicity against a cervical cancer cell line (CaSki), breast adenocarcinoma (MCF7) and glioblastoma cell line (SNB-19) at low micromolar concentrations. Polyhydroxystilbenes **11** and **12** showed the best potency against adenocarcinoma cells (GI_50_ = 7–8 μM). The inhibition activity towards cancer cells was comparable to those of the standard drug doxorubicin. The available from *R. tataricum* secondary metabolites may serve as new leads for the discovery of anticancer drugs.

## 1. Introduction

The genus *Rheum* (rhubarb, Polygonaceae) includes over 60 species, which are commonly known as edible plants in Asia, Europe and other regions of the world. The most important three species in the genus *Rheum*, *R. palmatum* L., *R. tanguticum* Maxim., and *R. officinale* Baill., are widely used for medicinal (roots and rhizomes), functional food (aerial parts) and cosmetic purposes (aerial parts) [1,2] Wild rhubarb species are mainly distributed in Asian countries such as China, India, Nepal, Korea, Bhutan, Pakistan, Turkey, Iran, Russia and Kazakhstan [2,3]. In both traditional Chinese and Tibetan medicine, the genus *Rheum* is credited with various pharmacological effects, such as treating liver, kidney, uterus and bladder diseases, hiccups, diarrhea, constipation, insect bites and relapsing fevers [1,3]. An analysis of the above data showed that research on rhubarb has made great progress in recent years. Several phytochemical studies have demonstrated that the main structures present in different species of this genus are anthraquinones, phytosterol, anthrones, different flavonoid and phenolic glycosides, stilbenes, lignans, tannins and carbohydrates [3,4,5]. Environmental factors have a significant impact on the levels and components of active pharmaceutical ingredients in rhubarb [6]. A chemical analysis of different rhubarb species showed that species of sect. deserticola contained higher amounts of glucose gallates, chromones and stilbenes and very small amounts of free anthraquinones and anthraquinone glycosides [7]. Differences in the chemical composition of different *Rheum* species may result in different biological activities. These results may be useful not only for elucidating the taxonomic relationships between species but also for assessing the quality of rhubarb to ensure its effective and safe use in the clinic.

The rhubarb species *R. tataricum* L.fil. has a limited habitat, occupying the territory of dry steppes and deserts of Central Asia (Astrakhan region in Russia, Central, Southern and Western Kazakhstan, the western region of China Xinjiang and Afghanistan), and it belongs to a group of rhubarb species with high adaptability to semi-desert and desert conditions, where both morphological changes (large leaf area, deformed small stems and deep roots) and biochemical features ensure the survival of the plant. Despite previously completed successful intensive morphological studies of *R. tataricum*, biochemical studies and the characterization of its chemical composition have been very limited and are still quite fragmentary [8]. Important components of *R. tataricum* collected in the desert zones of Kazakhstan are flavonoids [9], catechins and tannins [10], stilbenes (resveratrol) [11], and free and bound carbohydrates [12]. Analyses have been conducted using the LC-MS/MS technique to also quantify 2-aryl-4H-chromen-4-ones and quinic, gallic and chlorogenic acids [13]. For the first time, in the framework of this work, a sample growing in Syugaty Valley, belonging to another floristic region—Zailiysky Alatau—was studied. The environmental conditions of this territory are significantly different: it comprises gravelly soils of the foothill plain and is devoid of sandy areas and a different plant community predominates. In this region, *R.* *tataricum* mainly forms pure thickets, while previously studied populations, for example, in the Aral Sea region and Balkhash region, grow in communities with a predominance of saxaul on takyr-like or sandy soils. Thus, the chemical composition of *R.* *tataricum* L., growing in the conditions of Zailiyskiy Alatau, has not been previously studied (in these growing conditions and this floristic area).

The aim of this study was to examine the constituents of extractive compounds and isolation of phenolic compounds, 2-*O*-β-*D*-glucopyranosides of (*R*)- and (S)-4-(4-hydroxyphenyl)-2-butanol (rhododendrin **1** and *epi*-rhododendrin **3**), gallic acid **2**, the aglycones of compounds 1 and 3, 4-(3-hydroxybutyl)phenols ((2*R*)-rhododendrol **4** and (2*S*)-rhododendrol **5**), gallotannin β-glucogallin **6**, chlorogenic acids (3,5-di-*O*-caffeoylquinic acid **7** and 3-*O*-(*p*-coumaroyl)-5-*O*-caffeoylquinic acid **8**, 4-(4-hydroxyphenyl)-2-butanone (raspberry ketone) **9**, and polyhydroxystilbenes (rhaponticin **10**, desoxyrhaponticin **11** and resveratroloside **12**) from the ethanol extract of roots and rhizomes of *R. tataricum* L., growing in the conditions of Zailiyskiy Alatau. Additionally, we also analyzed the content of the most abundant compounds: rhododendrin **1** and gallic acid **2** of the *TBME* (*tert*-butyl methyl ester) and *EtOAc* fractions of *R. tataricum* ethanol extract by HPLC. One of the objectives of the present paper was to investigate the cytotoxicity in vitro of extract fractions and also of the accessible compounds **1**, **4**, **6**, **9**, **11**, and **12** from *R. tataricum* (the MTT test).

## 2. Results and Discussion

The plant material was extracted by 95% ethanol. The obtained extract (yield 18.2%) was fractioned with hexane, *TBME* (*tert*-butyl methyl ester) and *EtOAc*, which allows the separation of the reserve sugars, and we also isolated glycoside-containing compounds [14]. The chemical constituent of the hexane fraction (yield 1.3%) of the ethanol extract containing free sterols, their esters and fatty acids (by GC-MS) was not analyzed in detail. The yield of the *TBME* soluble fraction amounted to 10% of the whole extract. The yield of the *EtOAc* soluble fraction of the extract was approximately 31% of the extract’s weight. The residue of the ethanol extract (yield 56%) was hydrolyzed to identify gallic acid **1**.

### 2.1. Analysis of Components of TBME and EtOAc Fractions of Ethanol Extract of R. Tataricum by HLPC Method

The chromatographic profiles of *TBME and EtOAc* fractions of *R. tataricum* ethanol extract were analyzed by HPLC (ZORBAX SB-C18 column) (Figures S1 and S2). The chromatographic profiles of *TBME* and *EA* fractions showed almost identical substances, but the ratio of the main compounds differed.

**Figure 1:**
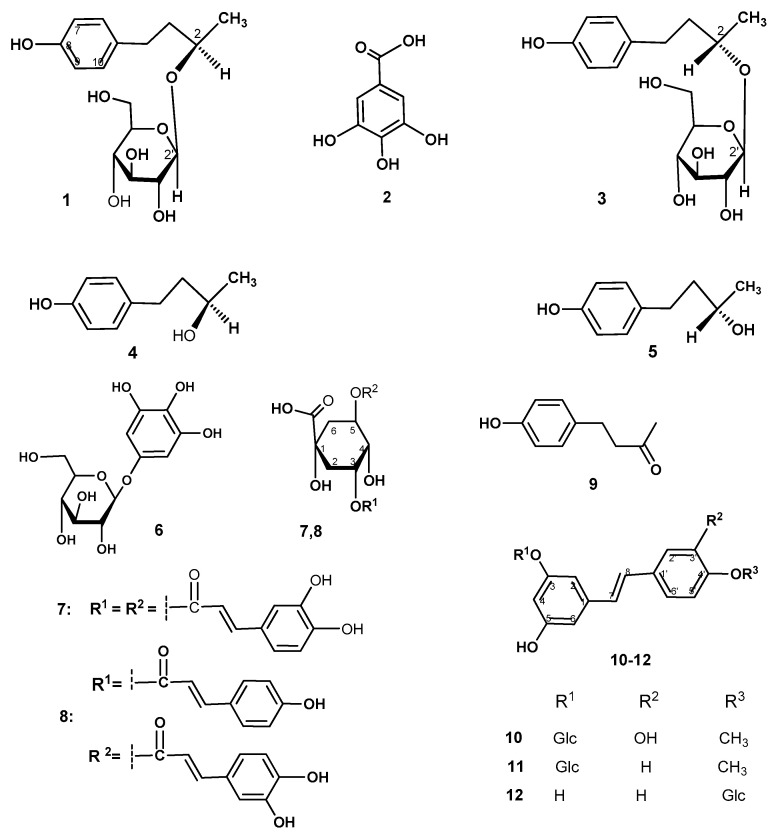
Structures of isolated compounds **1**–**12**.

The main compound of the studied *MTBE* and *EtOAc* fractions of the extract is the compound with a retention time of 12.7 min. The structure of this compound, *O*-β-*D*-glucopyranoside of *R*-(4-hydroxyphenyl)-2-butanol (**1**) (Figure 1), was established by the NMR method. In addition, gallic acid (**2**) (a retention time of ⁓4.6 min) was identified in the studied extracts. The content of compound **1** and gallic acid **2** in the MTBE extract was 581 and 16 mg/g, respectively. The content of compounds **1** and **2** in the EtOAc extract was 160 and 17 mg/g, respectively.

### 2.2. Separation of TBME and EtOAc Tractions of Ethanol Extract of R. tataricum and Structural Elucidation of Isolated Compounds

#### 2.2.1. Separation of TBME Fraction

The *TBME* fraction of the ethanol extract of *R. tataricum* (2.95 g) was subjected to column chromatography over silica gel using gradient CHCl_3_–EtOH (100:1→5:1) to yield five fractions (A–E). An analysis of the obtained fractions by ^1^H NMR spectra showed that fractions D and E (1.93 g) contain 4-(hydroxyphenyl)butanoids as the main compounds. By the repeated column chromatography of the combined fractions on silica gel, diastereomeric mixtures of (2*R*)- and (2*S*)-*O*-β-*D*-glucopyranoside of 4-(4-hydroxyphenyl)-2-butanols **1** and **3** (0.72 g, a ratio ⁓5:1 by the ^1^H NMR spectrum) (eluted with CHCl_3_–EtOH (10:1→5:3) and a diastereomeric mixture of the corresponding (2*R*)- and (2*S*)-4-(4-hydroxyphenyl)-2-butanols **4** and **5** (0.015 g) were isolated. The further separation of the fraction of glycosides **1** and **3** by TLC plate chromatography afforded pure compound **1** (by HPLC >95%, Figure S3). The repeated column chromatography of fraction B (0.31 g) (eluent CHCl_3_–EtOH (100:1→20:1) allowed the isolation of pure gallotannin 1-*O*-galloyl-β-*D*-glucose (β-glucogallin) **6** (0.132 g, yield ⁓4.5% from fraction weight). By the repeated column chromatography of fraction C (0.47 g) (eluent CHCl_3_–EtOH (100:0→100:4) chlorogenic acids 3,5-di-*O*-caffeoylquinic acid **7** (0.017 g) and 3-*O*-(*p*-coumaroyl)-5-*O*-caffeoylquinic acid **8** (0.011 mg) were isolated. The overall isolated yield of isolated compounds **1**–**8** was approximately 16% of the *TBME* fraction weight and 1.7% of the ethanol extract weight.

#### 2.2.2. Separation of *EtOAc* Fraction

The separation of a sample of the EtOAc fraction of the ethanol extract of *R. tataricum* (2.09 g) by column chromatography (eluent 100:0→2:1) and preparative thin-layer chromatograph, allowed the isolation of gallotannin β-glucogallin **6** (0.029 g). The Caffeoyl- and *p*-coumaroylquinic acid-rich fraction (^1^H NMR analysis) was further purified using additional chromatographic columns on silica gel to give the same 3,5-di-*O*-caffeoylquinic acid **7** (0.015 g) and 3-*O*-(*p*-coumaroyl)-5-*O*-caffeoylquinic acid **8** (0.012 g). From phenylbutanoid fraction glycosides of (4-hydroxyphenyl)-2-butanols **1** and **3** (0.13 g, ratio ⁓5:1 by ^1^H NMR spectrum) and (2*R*,*S*)-(4-hydroxyphenyl)-2-butanols **4** and **5** (0.042 g), 4-(4-hydroxyphenyl)butan-2-one (raspberry ketone) **9** (0.016 g) and three known stilbenes, rhaponticin **10** (0.012 g), desoxyrhaponticin **11** (0.021 g) and resveratroloside **12** (0.027 g) (Figure 1) were isolated. The overall isolated yield of compounds **1**, **3**–**12** was approximately 15% from the *EtOAc* fraction weight and 1.01% from the ethanol extract weight.

**Figure 2:**
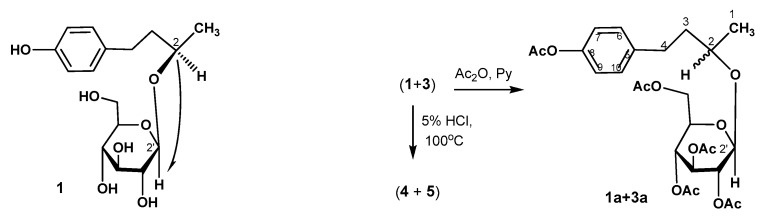
Diagnostic correlation observed in the long-range C-H COSY (→) for compound **1** and acid hydrolysis and acetylation of glycosides **1** and **3**.

The structure of all isolated compounds was confirmed on the basis of spectral and analytical data. The ^1^H NMR spectra of 1 exhibits a pair of doublet signals at δ 6.63 and 6.98 ppm (for diastereomer 3 – at 6.61 and 7.02 ppm), which represents the presence of a p-substituted benzene ring. A signal appeared at δ 1.20 (d, *J* = 6.8 Hz) (for **1**) and 1.24 ppm (d, *J* = 7.0 Hz) (for **3**) (∆δ 0.04 ppm), which are assignable to the protons of a methyl group. The acetylation of a mixture of glycosides **1** and **3** afforded a mixture of pentaacetates **1a** and **3a**, the ratio (⁓4.4:1) (Figure S3) of which was the same as in the glycoside mixture (**1** and **3**) (Figure 2). A characteristic feature of the NMR spectra of acetates is an increase in the difference in chemical shifts of the doublets of methyl groups (δ 1.10, 1.24 ppm ∆δ 0.14 ppm). The location of glycoside residue in compounds **1** and **3** was established based on the analysis of the ^13^C-H (HMBC) correlation spectrum by the presence of a cross-peak of the C-2 carbon atom with the H-2 protons of the glycoside fragment (Figure 2). These data indicate the location of the glycoside fragment at the C-2 atom in structures **1** and **3**. The acid treatment of the mixture of glycosides **1** and **3** by 5% HCl solution yielded a mixture of aglycones **4** and **5**. The aglycone of compound **1**, (*R*)-4-(3-hydroxybutyl)phenol named (-)-rhododendrol **4**, was found to co-occur in many species. Glycoside **1**, with the name rhododendrin, along with its aglycone (-)-rhododendrol **4**, was isolated for the first time from the leaves of *Rhododendron chrysanthurn* Linn. [15]. Later, a similar glycoside, named betuloside, was isolated from the bark of the birch sp. *Betula alba* L. [16], *B. pendula* (with (-)-rhododendrol **4**) [17] and *Taxus baceata* [18]. The aglycone (+)-(2*S*)-rhododendrol **5** and its glucoside *epi*-rhododendrin **3** were isolated in 1978 from *Acer nikoense* Maxim. [19]. Characteristically, compounds from the inner bark of *Betula pubescens*, both rhododendron **1** and *epi*-rhododendrin **3,** were isolated [20]. More recently, compound **5** and *epi*-rhododendrin **3** were isolated from roots and rhizomes of *Rheum maximowiczii* [21]. These results establish the stereochemical assignments to different samples of this glucoside isolated from different plants. From the X-ray diffraction studies, the absolute configuration at the stereocenter in the aglucone portion of compound **1** has been found to be *R* [22]. ^1^H and ^13^C NMR investigations gave identical results for compounds **4** and **5**, but the respective specific rotations rotations ([α]_D_^26 −^16.2 (*c* 0.5, EtOH) for **4** and [α]_D_^26^ +12.8 (*c* 0.5, EtOH) for **5**) differed. From these observations, **5** is suggested to be (+)-(2*S*)-rhododendrol, the enantiomer of (2*R*)-4-(3-hydroxybutyl)phenol (-)-4, and **3** is (*S*)-4-hydroxyphenyl-2-butanol 2-*O*-β-*D*-glucopyranoside (*epi*-rhododendrin).

From the both *TBME* and *EtOAc* fractions of *R. tataricum* ethanol extract, β-glucogallin **6** was isolated (Figure 1). Compound **6** is the simplest hydrolysable tannin, a well-known plant metabolite and major biosynthetic precursor for the production of larger more complex hydrolysable tannoids. Gallic acid glycoside **6** was isolated from Chinese [23] and Japanese rhubarbs [24].

Chlorogenic acids are generally involved in plant disease resistance responses to biotic or abiotic stress [25]. Two quinic acid derivatives with two cinnamoyl substituents, 3,5-di-*O*-caffeoylquinic acid **7** and 5-*O*-caffeoyl-3-*O*-*p*(coumaroyl)quinic acid **8**, were isolated by the column chromatography of samples of the ethanol extract of *R. tataricum* (Figure 1). These isolated compounds belong to hydroxylated phenylpropanoids and were isolated from *R. tataricum* for the first time. Natural and synthetic chlorogenic acids are of interest for their potent anti-inflammatory, antioxidant, and hepatoprotective properties [25,26]. The presence of quinic and chlorogenic acid in *R. tataricum* was identified by LC-MS/MS analysis [13].

The separation of the EtOAc fraction of *R. tataricum* extract allowed the isolation of a phenylbutanoid compound 4-(4-hydroxyphenyl) butan-2-one (raspberry ketone) **9**. Previously, this secondary metabolite was isolated from *R. tanguticum* [27] and *R. officinale* [28]. This compound has started to attract growing attention in recent years due to its potential weight loss function [29].

A characteristic future of the studied *R. tataricum* extract was the presence of stilbene compounds. By the column chromatography on silica gel of the EtOAc fraction of *R. tataricum* extract, three stilbenoids, rhaponticin **10**, desoxyrhaponticin **11** and resveratroloside **12**, were isolated. Stilbenes were isolated from various rhubarb species [30]. These stilbenoid metabolites were isolated from *R. tataricum* for the first time. Structures of compounds **10**–**12** (Figure 1) were elucidated on the basis of detailed spectroscopic analyses. The structure of stilbene **11** was established by X-ray analysis data.

**Figure 3:**
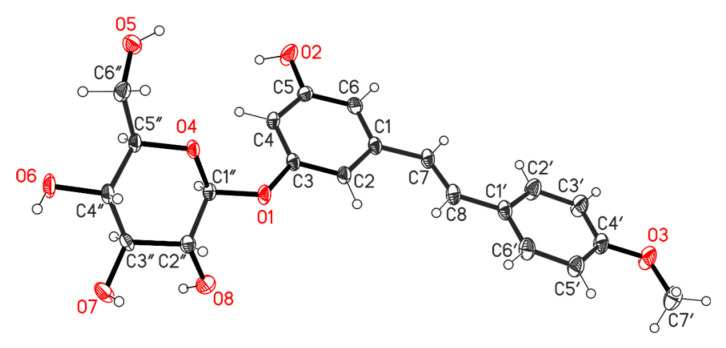
The molecular structure of desoxyrhaponticin (**11**). The thermal ellipsoids are drawn at the 30% probability level (CCDC 2193450).

The molecular structure of compound **11** is shown in Figure 3. The bond lengths and bond angles are the same as the statistical means and are very close to the corresponding ones found in the Cambridge structural database for the structure of rhaponticin **10** [31]. The aryl rings are perfectly planar, and the dihedral angle between ring (C1–C6) and ring (C1′–C6′) is 10.5(2)°. The chair conformation of the glucose moiety can be characterized by C1″ and C4″ atoms, deviations from the plane of C2″, C3″, C5″, and O4 atoms in the opposite sites by −0.658(3) Å for C1″ and 0.679(4) Å for C4″. The glucose moiety and phenyl ring (C1–C6) are rotated by 41.6(1)°, with respect to each other.

The packing diagram of the crystal structure is shown in Figure S20. It can be seen that C–H**^…^**O hydrogen bonds play a crucial role in directing the molecular assembly of **11** in the solid state. Details of these hydrogen bond lengths and bond angles are given in Table S1.

The obtained experimental data revealed that the roots of *R. tataricum* L.fil. were very rich in the (2*R*)- and (2*S*)-*O*-β-*D*-glucopyranoside of *R*-(4-hydroxyphenyl)-2-butanols (compounds **1** and **3**, and the overall isolated yield was 1.1% and the ration of rhododendrin **1** to *epi*-rhododendrin **3** was about 5:1). Other substance groups included gallotannin gallic acid glycoside **6**, polyhydroxyphenylpropanoids **7** and **8**, raspberry ketone **9** and stilbenes **10**–**12**. The isolated yield of the simplest hydrolysable tannin **6** reached 0.54%. The isolation of esters of quinic acid with dicaffeoyl- and 5-caffeoyl-3-(*p*-coumaroyl) substituents **7** and **8** was performed. These biologically important phenolic compounds are present in many plant species [32] but, from *R. tataricum* were isolated for the first time. Rhaponticin **10** is an important compound of rhubarb [33]. This compound is commonly present in species of sectio Rhapontica and therefore is examined as a major chemotaxonomical marker.

### 2.3. Cytotoxicity Assay

The high isolated yield of the whole ethanol extract from the root and rhizomes of *R. tataricum* and the ease of the isolation of some secondary metabolites from the rhizomes of *R. tataricum* L., growing in the conditions of Zailiyskiy Alatau, were the cause of our interest in investigating the cytotoxic activity of these extracts, both TBME and EtOAc fractions and isolated compounds. The extract and compounds **1**, **4**, **6**, **9**, **11** and **12**, as well as a mixture of acetates **1a**–**3a**, were screened for their in vitro cytotoxic and growth inhibitory activities against a panel of human cancer cell lines (three cervical tumor cell lines (C-33 A, CaSki, and HeLa), breast cancer cells (MCF-7), prostate cancer cells (DU-145) and two glioblastoma cell lines (T98G and SNB-19)). Normal epithelial VERO cells were used as the non-cancer control. Cell viability was assessed by the 3-[4,5-dimethyl-thiazol-2-yl]-2,5-diphenyltetrazolium bromide (MTT) assay [34]. Cytotoxicity was determined by measuring the concentration inhibiting human tumor cell viability by 50% (GI_50_). Doxorubicin, which is one of the most effective anticancer agents, was used as the reference drug in this study. The results are listed in Table 1. A comparison of the results revealed the following. The extract, its fractions and isolated compounds **1**, **4**, **6**, **9**, **11** and **12** were non-toxic for VERO cells (GI_50_ > 83 μM) relative to doxorubicin (GI_50_ = 8 ± 1.8 μM) (Table 1). The extract possessed low cytotoxicity towards the studied cancer cell lines. In the group of isolated compounds, the activity of 4-hydroxyphenylbutanoids **1** and **4** draw our attention. An analysis of the data on cytotoxicity toward cervical cancer cells (C33 A, CaSki and Hela) revealed selective cytotoxicity towards CaSki cell lines for rhododendrin **1** and rhododendrol **4** compared to the activity of doxorubicin. Compounds **1** and **4** also exhibited anticancer activity towards breast adenocarcinoma cell line MCF-7 in the micromolar range. Acetylation evidently led to this activity decreasing; for example, acetylated compound (**1a** + **3a**) lost its activities.

Isolated stilbenes desoxyrhaponticin **11** and resveratroloside **12** possessed the highest degree of cytotoxicity towards breast adenocarcinoma cell line MCF-7. The cytotoxicity of compounds **11** and **12** was comparable with that of doxorubicin. Stilbenes **11** and **12** exhibited cytotoxicity towards glioblastoma cancer cell lines, with the higher cytotoxicity towards SNB-19 cell lines. Notably, resveratroloside **12** demonstrated an increase in potency on the SNB-19 cell lines and was comparably as active in this assay as the drug doxorubicin. These data were in agreement with the reported data about the anticancer activity of natural polyhydroxystilbenes in human cancer cells [33,35,36]. These compounds represent new alternatives for the development of cancer therapeutics [37,38]. Of interest are previous results, which have shown the cytotoxic effects of (*R*,*S*)-4-(4-hydroxyphenyl)-2-butanols **4** and **5** [39]. Moreover, rhododendrin **1** exhibited significant analgesic actions in mice and anti-inflammatory actions in rats [40]. Rhododendrin **1** was also considered as a hepatoprotective constituent of *Taxus baccata* [22]. *epi*-Rhododendrin **3** and (+)-rhododendrol **5** suppressed NO production by activated macrophages in vivo and have a potentiality to be anti-inflammatory drugs [41]. The data obtained at this stage show the promise for the further mechanistic, biological and toxicological study of the *R. tataricum* L.fil. extract and its secondary metabolites.

## 3. Materials and Methods

### 3.1. Chemistry

#### 3.1.1. General Information

Melting points were determined on the thermosystem Mettler Toledo FP900 (Columbus, OH, USA). NMR spectra were acquired on Bruker Avance-400 (^1^H: 400.13 MHz, ^13^C: 100.78 MHz) or Bruker DRX 500 (^1^H: 500.13 MHz, ^13^C: 125.76 MHz) spectrometers (Bruker Corporation, Karlsruhe, Germany). Deuterochloroform (CDCl_3_), a mixture of (CDCl_3_+CD_3_OD, 1:1, *v*/*v*) or ((CD_3_)_2_SO), was used as a solvent. By using CDCl_3_ as a solvent, the residual CHCl_3_ (δ_H_ = 7.24 ppm) and CDCl_3_ (δ_C_ = 76.8 ppm) were employed as internal standards, and by using (CD_3_)_2_SO as a solvent, the residual of DMSO (δ_H_ = 2.51 ppm) was employed as the internal standard. NMR signal assignments were carried out with the aid of a combination of 1D and 2D NMR techniques that included ^1^H, ^13^C, COSY, HSQC and HMBC spectra. NMR (^1^H and ^13^C) spectra of **1**, (**1a** + **3a**), **6**, **9**, **11**, and **12** are given in the Supplementary Materials. IR spectra were recorded by means of the KBr pellet technique on an Avatar 360 ESP spectrometer (Termo Nicolet, Madison, WI, USA). The UV spectra were obtained on a Cary 60 UV Vis spectrometer (Agilent Technologies, Santa Clara, CA, USA). Elemental analysis was carried out on a Carlo-Erba 1106 CHN-analyzer. GC-MS was performed using an Agilent 7890B/5977A instrument with an Agilent 7890B gas chromatograph and an Agilent 5977A (EI, 70 eV) mass-selective detector(Agilent Technologies, Santa Clara, CA, USA). Mass spectra were recorded on a (Thermo Fisher Scientific, Waltham, MA, USA) in full-scan mode (15–500 *m*/*z*, evaporator temperature 150–280 °C, 70 eV electron-impact ionization). The specific rotation values [α]_D_ were determined on a PolAAr 3005 polarimeter (Optical Activity Ltd., Huntingdon, UK). The X-ray structural study of compound **11** was performed on a Bruker KAPPA APEX II diffractometer with a two-dimensional CCD detector (MoKα radiation with a graphite monochromator and ω-φ-scanning).

HPLC analysis was performed on an Agilent LC 1100 chromatograph (Agilent Technologies, Santa Clara, CA, USA) equipped with a quaternary pump, an autosampler, and a diode array detector. The chromatographic conditions were as follows: a ZORBAX SB-C18 column (4.6 × 150 mm, 5 μm) (Agilent Technologies) was used; a mobile phase consisting of methanol/0.1% (*v*/*v*) trifluoroacetic acid in an H_2_O gradient was utilized; the methanol percentage in the gradient was 20–95% (minutes 0–30); the flow rate was 0.6 mL/min; the injection volume was 2 μL; and the detection was performed simultaneously at wavelengths 254, 280 and 320 nm. Gallic acid from Sigma-Aldrich (St. Louis, MI, USA) was used for the quantification of gallic acid. The analytical HPLC of compounds **1**, **4**, **9**, **11**, and **12** was given in Supplementary Materials.

Extractive compounds were isolated by column chromatography on silica gel (0.063–0.200 mm, Acros or 60–0.120 mm, Merck KGaA, Darmstadt, Germany) and eluated with chloroform, chloroform–ethanol (*v*/*v* = 100:0; to 0:l); chloroform–methanol (*v*/*v* = 10:0; to 1:10), and EA–acetone (*v*/*v* = 5:1; to 0:1). Preparative TLC was carried out on glass plates (30 × 30 cm with 2 mm thick sorbent layer; silica gel F_254_ 35–70 μm, Across-Organics (BVBA, Belgium) or on silica gel 60 F_254_ plates (Qingdao Marine Chemical, Inc., Jiaolai town, Qingdao, China). The purity of separation was monitored by TLC on Silufol UV_254_ plates using various solvent systems, and spots were visualized by detection under UV light or by treatment with iodine vapor. The solvents ethanol, hexane, *t*-butylmethyl ether (*ТBМE*), chloroform (CHCl_3_), methanol (MeOH), ehter and ethyl acetate (*EtOAc*) were purified according to standard methods and distilled immediately before use.

#### 3.1.2. Plant Material

The underground part of *Rheum tataricum* L.fil. was collected during the vegetation– early-budding phase on 12 April 2022, in the Syugatin Valley, 8 km east of the village of Kokpek, located in the Enbekshikazakh District of the Almaty Region, the Republic of Kazakhstan (GPS coordinates: N 43°25′16.7″, E 78°44′47.8″, at an altitude of 1173 m above sea level). The herbarium specimen of *Rheum tataricum* L.fil. was collected and identified by Zh.Zh. Karzhaubekova and R.B. Arysbayeva and the species was identified by N.G. Gemejiyeva and is stored in the Herbarium (AA) of the Institute of Botany and Phytointroduction (Almaty, Republic of Kazakhstan) under voucher number 2197/25.

#### 3.1.3. Preparation of Extracts of *R. tataricum*

The dry rhizomes (the underground part) (162 g) of *R. tataricum* were extracted with 96% ethanol (3 × 1700 mL EtOH) by stirring and heating to 80 °C for 6 h. The extracts were combined and evaporated under vacuum to obtain 29.57 g (18% of dry rhizomes). The dry residue was further suspended in hexane (300 mL) to remove lipophilic compounds. The yield of hexane extract was 0.38 g (1.3% from the crude extract). Then, the residue was extracted with tert-butyl methyl ester (*TBME*) (2000 mL, 50 °C, 8 h). After concentration on a rotary evaporator, the *TBME* fraction (2.95 g, yield 10% from the crude extract, or 1.8% from dry rhizomes) was obtained. The extraction of the residue ethanol extract was performed with EtOAc (1500 mL, 80 °C, 5 h). The EtOAc soluble portion was collected separately and concentrated with a rotary evaporator to obtain 9.14 g of the *EtOAc* fraction (yield 30% from the crude extract, or 5.6% from dry rhizomes).

The treatment of the residue of the extract (1 g) with acetone (20 mL) at room temperature (12 h), filtration, evaporation and trituration with ether afforded a white powder of gallic acid **2** (m.p. 250 °C). A portion of the residue of the extract (1.84 g) was treated with acetone (100 mL) and treated under stirring with (CH_3_)_2_SO_4_ (5 mL) and K_2_CO_3_ (2 g). The mixture was stirred under reflux for 4 h and then cooled and filtered. After evaporation, methyl 3,4,5-trimethoxybenzoate (426 mg) was isolated as a yellowish solid. M.p. 80–82 °C (ether). ^1^H NMR (500 MHz, (CD_3_)_2_SO, δ, ppm): 3.33 (3H, s, OCH_3_-4), 3.94 (3H, s, OCH_3_-7), 3.97 (6H, s, OCH_3_-3,5), 7.11 (2H, s, H-2,6).

#### 3.1.4. Acetylation of Glycosides (**1** + **3**) (From TBME Fraction of Ethanol Extract of *R. tataricum*)

A fraction of glycosides (**1** + **3**) ([α]_D_^25^ −20.8 (*c* 0.8, EtOH) (0.38 g) was treated with a solution of Ac_2_O (1.5 mL) and pyridine (1.5 mL). The obtained solution was stirred at room temperature for 25 h, then poured on ice and extracted with ethyl acetate (50 mL). The evaporation of the solvent and treatment of the residue with ether afforded a precipitate of pentaacetates (**1a** + **3a**) (0.59 g). M.p. 109–112 °C; [α]_D_^25^ −26.8 (*c* 0.8, CHCl_3_). ^1^H NMR (500 MHz, (CDCl_3_, δ, ppm): 1.10 (d, *J* = 6.8 Hz, CH_3_- of **3a**), 1.24 (d, *J* = 6.8 Hz, CH_3_- of **1a**) (3a:1a⁓1:4.4), 1.60–1.72 (m, H-3), 1.78–1.84 (m, H-3), 1.98, 2.0, 2.03, 2.04 (all s, 4 × CH_3_-OAc), 2.266, 2.268 (both s, Ac, ratio ⁓5:1), 2.48–2.57 (m, H-4), 2.88–2.95 (m, H-2′), 3.58–3.76 (m, H-5′,4′), 4.09 (dd, *J* = 12.0, 5.2 Hz, H-6′), 4.21 (dd, *J* = 12.0, 2.2 Hz, H-6′), 4.48 (d, *J* = 6.8 Hz, H2- of **3a**), 4.54 (d, *J* = 6.8 Hz, H2-of **1a**), 4.94–5.10 (m, H-3′), 5.16–5.21 (m, H-1′), 6.93 (d, *J* = 8.0 Hz, H-7,9 of **3a**), 6.97 (d, *J* = 8.0 Hz, H-7,9 of **1a**), 7.13 (d, *J* = 8.0 Hz, H-6,10 of **1a**), 7.17 (d, *J* = 8.0 Hz, H-6,10 of **3a**); ^13^C NMR (125 MHz, CDCl_3_, δ, ppm): 19.74, 20.45, 20.51, 20.60, 20.62 (4-CH_3_-Ac), 21.01, 21.64 (C-1), 30.52 (C3-3a), 30.77 (C3-1a), 38.25 (C4-3a), 38.50 (C4-1a), 61.94 (C6′-3a), 62.04 (C6′-1a), 68.43, 68.46 (C-4′), 71.40 (C2-3a), 71.62 (C2-1a), 72.85 (C2′-1a), 73.45 (C2′-3a), 76.63 (C-3′), 77.55 (C-5′), 99.01(C1′-3a), 101.05 (C1′-1a), 121.11 (C-7,9-3a), 121.40 (C-7,9-1a), 129.08 (C-6,10-1a), 129.39 (C-6,10-3a), 139.24 (C5-1ª), 139.58 (C5-3a), 148.59, 148.71 (C-8), 169.10, 169.18, 169.21, 169.55, 170.25 (5 × C=O); HR-MS, *m*/*z* (I*_rel._*, %): 538 (0.5), 497 (3), 496 (10), 464 (10), 332 (3), 331 (13), 191 (5), 190 (17), 169 (25), 149 (41), 148 (61), 126 (32), 107 (52), 98 (17), 97 (20), 85 (15), 73 (21), 69 (19), 60 (38), 57 (29), 43 (100); calcd for C_26_H_34_O_12_: 538.2045; found [M − H]^+^ *m*/*z*: 538.2044.

#### 3.1.5. Hydrolysis of Glycosides (**1** + **3**)

A solution of glycosides (**1** + **3**) ([α]_D_^25^ −20.8 (*c* 0.8, EtOH) (0.26 g) in MeOH (20 mL) was treated with 5% HCl (5 mL) and the mixture was refluxed for 2 h. After cooling, the mixture was treatment with some drops of 5% KOH (to pH ⁓6.0) and the main solution was evaporated in vacuo. The residue was treated with EtOAc (100 mL) and the organic layer was washed with water, dried over MgSO_4_, filtered and evaporated. The obtained crude product (0.16 g) was subjected to column chromatography (20 g silica gel, eluted with CHCl_3_→CHCl_3_-EtOH, 50:1). By the repeated chromatography of the first fraction on silica gel (eluted by abs. CHCl_3_) and crystallization with EtOAc, (-)-rhododendrol **4** (0.061 g) was isolated. From the second fraction (eluting it with CHCl_3_-EtOH, 100:1→50:1, and evaporating and triturating the residue with ether), (+)-rhododendrol **5** (0.018 g) was isolated.

#### 3.1.6. Structure Determination of Isolated Compounds

*(R)-4-(4-Hydroxyphenyl)-2-butanol 2-β-D-glucopyranoside* (*Rhododendrin*) (**1**). White solid. M.p. 183–186 °C (ether). [α]_D_^26^ −42.7 (*c* 0.5, EtOH); (lit.: M.p. 188–189 °C. [α]_D_ −46 (*c* 1.0, EtOH) [20]; M.p. 189 °C. [α]_D_ −38 (*c* 0.5, MeOH) [40]); ^1^H NMR (400 MHz, (CD_3_)_2_SO, δ, ppm): 1.19 (3H, d, *J* = 6.8 Hz, CH_3_-1), 1.55–1.60 (1H, ddd, *J* = 13.2, 6.7, 2.0 Hz, H-3), 1.50–1.75 (1H, ddd, *J* = 13.2, 7.0, 2.0 Hz, H-3), 2.84 (2H, m, H-4), 2.93 (1H, dd, *J* = 8.0, 8.2 Hz, H-2′), 3.04 (1H, m, H-5′), 3.14 (1H, m, H-4′), 3.34 (3H, br.s, OH), 3.34 (1H, dd, *J* = 12.0, 5.2 Hz, H-6′), 3.41 (1H, dd, *J* = 12.0, 2.2 Hz, H-6′), 3.64 (1H, dd, *J* = 12.0, 8.2 Hz, H-3′), 3.79 (1H, m, H-2), 4.16 (1H, d, *J* = 8.2 Hz, H-1′), 5.03 (2H, br.s, OH), 6.63 (2H, d, *J* = 7.8 Hz, H-7,9), 6.98 (2H, d, *J* = 7.8 Hz, H-6,10); ^13^C NMR (101 MHz, (CD_3_)_2_SO, δ, ppm): 21.74 (C-1), 29.77 (C-4), 38.50 (C-3), 61.12 (C-6′), 70.13 (C-4′), 73.66 (C-2′), 74.62 (C-2), 76.68 (C-3′), 76.80 (C-5′), 102.79 (C-1′), 115.08 (C-7,9), 129.17 (C-6,10), 131.93 (C-5), 155.64 (C-8); IR (KBr, ν, cm^−1^): 3430, 3225 (OH), 2988, 1605, 1505, 1490 (C=C), 1158 C-O), 1110, 1062, 1043, 1006 (glycosidic C-O); UV (MeOH) λ_max_ (lgε): 223 (4.79), 279 (4.56) nm; HR-MS, *m*/*z* (I*_rel._*, %): 328 (3, M^+^), 219 (10), 208 (5), 207 (17), 190 (23), 177 (27), 166 (28), 148 (32), 133 (22), 107 (100); calcd for C_16_H_24_O_7_: 328.1517; found [M − H]^+^ *m*/*z*: 328.1113.

*(S)-4-(4-Hydroxyphenyl)-2-butanol 2-β-D-glucopyranoside (epi-rhododendrin)* (**3**). White solid. M.p. 83–86 °C (ethylacetate), [α]_D_^26^ −13.6 (*c* 0.5, EtOH); (lit.: [α]_D_^20^ −15.5 (c 0.1, EtOH) [19]); ^1^H NMR (400 MHz, (CD_3_)_2_SO, δ, ppm): 1.24 (3H, d, *J* = 7.0 Hz, CH_3_-1), 1.59 (1H, ddd, *J* = 13.5, 7.0, 2.0 Hz, H-3), 1.83 (1H, ddd, *J* = 13.5, 7.0, 2.0 Hz, H-3), 2.64 (2H, m, H-4), 3.03 (1H, dd, *J* = 8.0, 8.2 Hz, H-2′), 3.14 (1H, m, H-5′), 3.25 (1H, m, H-4′), 3.49 (1H, dd, *J* = 12.0, 5.2 Hz, H-6′), 3.66 (1H, dd, *J* = 12.0, 2.2 Hz, H-6′), 3.71 (1H, dd, *J* = 12.0, 8.2 Hz, H-3′), 3.74 (1H, m, H-2), 4.22 (1H, d, *J* = 8.2 Hz, H-1′), 6.61 (2H, d, *J* = 7.8 Hz, H-7,9), 7.02 (2H, d, *J* = 7.8 Hz, H-6,10); ^13^C NMR (101 MHz, CDCl_3_, δ, ppm): 22.43 (C-1), 30.57 (C-4), 38.06 (C-3), 61.18 (C-6′), 72.07 (C-4′), 75.64 (C-2′), 76.46 (C-3′), 77.51 (C-2), 78.16 (C-5′), 104.08 (C-1′), 115.12 (C-7,9), 129.35 (C-6,10), 131.87 (C-5), 156.71 (C-8); IR KBr, ν, cm^−1^): 3419 (OH), 1612, 1518, 1500 (C=C), 1178 C-O), 1110, 1076, 1050, 1010 (glycosidic C-O); UV (MeOH) λ_max_ (lgε): 224 (4.81), 278 (4.11) nm; HR-MS, *m*/*z* (I*_rel._*, %): 328 (1, M^+^), 219 (10), 207 (16), 191 (4), 190 (20), 178 (9), 177 (32), 166 (41), 149 (15), 148 (48), 133 (28), 121 (17), 108 (23), 107 (100); calcd for C_16_H_24_O_7_: 328.1517; found [M − H]^+^ *m*/*z*: 328.1125.

*(R)-4-(4-Hydroxyphenyl)-2-butanol (-)-Rhododendrol)* (**4**). White needles. M.p. 81–84 °C (EtOAc), [α]_D_^26^ −16.2 (*c* 0.5, EtOH); (lit.: M.p. 81–83 °C, [α]_D_ −17.1 (*c* 2.0, EtOH,) [40]); ^1^H NMR (500 MHz, CD_3_OD+CDCl_3_, δ, ppm): 1.15 (3H, d, *J* = 6.5 Hz, CH_3_-1), 1.51 (1H, m, H-3), 1.65 (1H, ddd, *J* = 13.5, 6.8, 1.8 Hz, H-3), 2.51(1H, m, H-4), 2.60 (1H, m, H-4), 3.65 (1H, ddd, *J* = 12.0, 6.5, 5.0 Hz, H-2), 6.59 (2H, d, *J* = 7.8 Hz, H-7,9), 6.95 (2H, d, *J* = 7.8 Hz, H-6,10); ^13^C NMR (125 MHz, CDCl_3_+CDCl_3_, δ, ppm): 22.56 (C-1), 27.51 (C-4), 39.23 (C-3), 69.32 (C-2), 114.61 (C-7,9), 128.32 (C-6,10), 131.15 (C-5), 155.34 (C-8); IR (KBr, ν, cm^−1^): 3323 (OH), 1610, 1500 (C=C); UV (MeOH) λ_max_ (lgε): 221 (4.26), 278 (4.09) nm; HR-MS, *m*/*z* (I*_rel._*, %): 167 (1), (166 (9, M^+^), 148 (6), 133 (15), 108 (7), 107 (20), 46 (31), 45 (53), 31 (100); calcd for C_10_H_14_O_2_: 166.0988; found [M − H]^+^ *m*/*z*: 166.0989.

*(S)-4-(4-Hydroxyphenyl)-2-butanol (+)-Rhododendrol* (**5**). White solid. M.p. 75–78 °C (ether), [α]_D_^26^ +12.8 (*c* 0.5, EtOH); (lit.: M.p. 80–81 °C. [α]_D_ +13.6 (*c* 1.0, EtOH) [41]; [α]_D_ +15.5 (*c* 0.36, MeOH) [42]); ^1^H NMR (400 MHz, (CD_3_OD+CDCl_3_, δ, ppm): 1.20 (3H, d, *J* = 7.0 Hz, CH_3_-1), 1.55 (1H, m, H-3), 1.67 (1H, ddd, *J* = 13.5, 7.0, 1.8 Hz, H-3), 2.58–2.65 (2H, m, H-4), 3.61–3.74 (1H, m, H-2), 6.64 (2H, d, *J* = 8.2 Hz, H-7,9), 7.03 (2H, d, *J* = 8.2 Hz, H-6,10); ^13^C NMR (101 MHz, CDCl_3_, δ, ppm): 22.56 (C-1), 27.51 (C-4), 39.23 (C-3), 69.32 (C-2), 114.61 (C-7,9), 128.32 (C-6,10), 131.15 (C-5), 155.34 (C-8); IR (KBr, ν, cm^−1^): 3430 (OH), 1605, 1505, 1490 (C=C), 1185 C-O); UV (MeOH) λ_max_ (lgε): 223 (4.79), 279 (4.14) nm; HR-MS, *m*/*z* (I*_rel._*, %): 166 (20, M^+^), 148 (18), 133 (61), 108 (14), 107 (100); calcd for C_10_H_14_O_2_: 166.0988; found [M − H]^+^ *m*/*z*: 166.0991.

*1-O-Galloyl-β-D-glucopyranose (β-glucogallin)* (**6**). White powder. M.p. 210–212 °C (ether), [α]_D_^26^ −24.2 (*c* 0.2, CHCl_3_); [lit.: M.p. 214 °C (ethanol) [43]; [α]_D_ −23.4 (*c* 1.0, EtOH) [44]; [α]_D_ −8 (*c* 0.1, MeOH) [45]), ^1^H NMR (400 MHz, CDCl_3_+CD_3_OD, δ, ppm): 3.25–3.41 (4H, m, H-2′,3′,4′,5′), 3.64 (1H, dd, *J* = 12.4, 5.8 Hz, H-6′), 4.08 (1H, dd, *J* = 12.4, 1.5 Hz, H-6′), 4.61 (1H, d, *J* = 7.8 Hz, H-1′), 7.11 (2H, s, H-2,6); ^13^C NMR (101 MHz, CDCl_3_+CD_3_OD_,_ δ, ppm): 61.09 (C-6′), 66.30 (C-4′), 73.60 (C-2′), 76.46 (C-3′), 77.14 (C-5′), 103.87(C-1′), 104.80 (C-5), 125.53 (C-6), 138.29 (C-3), 152.01 (C-4), 166.35 (C-7).

*3,5-Di-O-Caffeoylquinic Acid* (**7**). Amorphous white powder. ^1^H NMR (400 MHz, (CDCl_3_+CD_3_OD, δ, ppm): 2.08 (4H, br.s, (H-2,6), 3.94 (1H, br.s, H-4), 5.32 (2H, br.s, (H-3,5), 6.27 (2H, d, *J* = 15.9 Hz, 2-H-2′), 6.89 (2H, d, *J* = 7.8 Hz, 2-H-8′), 7.01 (2H, d, *J* = 1.8 Hz, 2-H-5′), 7.05 (2H, dd, *J* = 7.8, 1.8 Hz, 2-H-9′), 7.58 (2H, d, *J* = 15.9 Hz, 2-H-3′) (OH protons not observed); ^13^C NMR (101 MHz, CDCl_3_+CD_3_OD, δ, ppm): 31.61 (C-6), 34.03 (C-2), 63.19 (C-4), 65.04 (C-3), 65.09 (C-5), 70.14 (C-1), 109.15 (2C-5′), 115.46 (2C-8′), 115.50 (2C-2′), 122.95 (2C-9′), 129.81 (2C-4′), 144.57 (2C-3′), 146.61 (2C-6′), 147.76 (2C-7′), 167.36 (2C-1′), 174.29 (C-7); IR (KBr, ν, cm^−1^): 3420, 3380 (OH), 1695 (C=O), 1605, 1525, 812, 745 (C=C); UV (MeOH) λ_max_ (lgε): 228 (4.05), 243 (3.41), 300 sh (3.05), 325 (3.91) nm. NMR, IR and UV spectral data match well with those reported previously [43]. Anal. calcd for C_25_H_24_O_12_, %: C, 58.14; H, 4.68; found, %: C, 58.45; H, 4.79.

*5-Caffeoyl-3-O-(p-coumaroyl)quinic Acid* (**8**). Amorphous yellowish powder. ^1^H NMR (400 MHz, (CDCl_3_+CD_3_OD, δ, ppm): 2.06 (4H, br.s, (H-2,6), 3.95 (1H, br.s, H-4), 5.16 (1H, br.s, (H-5), 5.26 (1H, br.s, (H-5), 6.27 (1H, d, *J* = 15.9 Hz, H-2′), 6.28 (1H, d, *J* = 15.9 Hz, H-2″), 6.82 (2H, d, *J* = 8.2 Hz, H-6″,8′′), 6.90 (1H, d, *J* = 7.8 Hz, H-8′), 7.01 (H, d, *J* = 1.8 Hz, H-5′), 7.05 (H, dd, *J* = 7.8, 1.8 Hz, H-9′), 7.40 (2H, d, *J* = 8.2 Hz, H-5″,9″), 7.60 (H, d, *J* = 15.9 Hz, H-3′), 7.62 (H, d, *J* = 15.9 Hz, H-3″) (OH protons not observed); ^13^C NMR (101 MHz, CDCl_3_+CD_3_OD, δ, ppm): 31.63 (C-6), 34.05 (C-2), 63.21 (C-4), 65.01 (C-3), 65.18 (C-5), 70.14 (C-1), 109.57 (C-5′), 115.46 (C-8′), 115.62 (C-2″),115.50 (C-2′), 115.74 (C-6″,8″), 122.95 (C-9′), 126.99 (C-4″), 129.80 (C-4′), 129.81 (C-5″,9″), 144.22 (C-3″), 144.57 (2C-3′), 146.61 (2C-6′), 147.76 (2C-7′), 157.76 (C-7″) 167.35 (C-1′), 167.51 (C-1″), 174.29 (C-7); IR (KBr, ν, cm^−1^): 3432 (OH), 1695 (C=O), 3080, 1600, 1523, 812, 763 (C=C); UV (MeOH) λ_max_ (lgε): 218 (4.32), 237 (3.53), 300 sh (3.15), 326 (3.75) nm. Anal. calcd for C_25_H_24_O_11_, %: C, 60.00; H, 4.83; found, %: C, 60.18; H, 4.67.

*4-(4-Hydroxyphenyl)butan-2-one* (**9**). White powder. M.p. 82–85 °C (ether); (lit.: M.p. 83.5–84.5 °C [46]); ^1^H NMR (500 MHz, CDCl_3_, δ, ppm): 2.11 (3H, s, CH_3_), 2.72, 2.82 (both 2H, A_2_B_2_ system, *J* = 7.6 Hz, CH_2_CH_2_-H-3,4), 5.35 (1H, br.s, OH), 6.73 (2H, d, *J* = 8.4 Hz, H-7,9), 7.01(2H, d, *J* = 8.4 Hz, H-6,10). ^13^C- NMR (125 MHz, CDCl_3,_ δ, ppm): 28.81 (C-4), 30.29 (CH_3_), 45.51 (C-3), 115.34 (C-7,9), 130.22 (C-6,10), 132.46(C-5), 154.19 (C-8), 209.89 (C=O); HR-MS, *m*/*z* (I*_rel._*, %): 164 (38, M^+^), 149 (6, [M-CH_3_]^+^), 121 (15 [M-Ac]^+^), 107 (100 [M-CH_2_Ac]^+^), 77 (51); calcd for C_10_H_12_O_2_: 164.0872; found [M − H]^+^ *m*/*z*: 164.0879.

*Rhaponticin (3,3′,5-Trihydroxy-4′-methoxystilbene-3-O-β-D-glucopyranoside)* (**10**). Yellowish needles; M.p. 248–250 °C (EtOH), [α]_D_ −55.2 (*c* 0.5; EtOH); (lit.: M.p. 246–248 °C (dil. acetone), [α]_D_ −56.3 (*c* 0.88, aq. acetone, 1:1) [30]); ^1^H NMR (400 MHz, CDCl_3_+CD_3_OD, δ, ppm): 3.26 (1H, m, H-2″), 3.38–3.46 (1H, m, H-3″,4″), 3.58 (1H, m, H-5″), 3.71 (1H, dd, *J* = 7.0, 11.8 Hz, H-6″), 3.78 (3H, s, 4′-OCH_3_), 3.93 (1H, dd, *J* = 3.8, 11.8 Hz, H-6″), 4.85 (1H, d, *J* = 7.0 Hz, H-1″), 6.35 (1H, t, *J* = 1.8 Hz, H-4), 6.58, 6.77 (each 1H, br.s, H-2,6), 6.85 (1H, d, *J* = 16.2 Hz, H-7), 7.02 (1H, d, *J* = 16.2 Hz, H-8), 6.84 (1H, d, *J* = 8.2 Hz, H-5′), 6.98 (1H, dd, *J* = 2,0, 8.2 Hz, H-6′), 7.08 (1H, d, *J* = 2.0 Hz, H-2′) (OH protons not observed); ^13^C NMR (101 MHz, CDCl_3_ +CD_3_OD, δ, ppm): 55.63 (OCH_3_), 60.91 (C-6″), 71.05 (C-4″), 74.31 (C-2″), 76.23 (C-5″), 77.48 (C-3″), 100.51 (C-1″), 102.84 (C-4), 105.02 (C-2), 107.21 (C-6), 112.11 (C-5′), 112.72 (C-2′), 118.61 (C-6′), 126.21 (C-7), 128.45 (C-8), 129.78 (C-1′), 139.14 (C-1), 146.43 (C-3′), 147.41 (C-4′), 158.08 (C-5), 158.71 (C-3); IR (KBr, ν, cm^−1^): 3456, 3340 (OH), 1610, 1583, 1510, 815, 723 (C=C), 1085, 1015 (sugar); UV (MeOH) λ_max_ (lgε): 220 (4.36), 302 (4.09), 324 (4.45); HRMS: (*I*_rel._, %): 420 (M^+^, 1), 258 (100, M^+^- Glc), 257 (32), 256 (18), 197 (26), 129 (16), 115 (16); calcd for C_21_H_24_O_9_: 420.1466; found [M − H]^+^ *m*/*z*: 420.1461.

*Desoxyrhaponticin (3,5-Dihydroxy-4′-methoxystilbene-3-O-β-D-glucopyranoside)* (**11**). Colorless plate. M.p. 227–230 °C (EtOH), [α]_D_ −56.6 (*c* 0.3, EtOH); (lit.: M.p. 226–228 °C (EtOH), [α]_D_ −50 ± 2 (*c* 0.2; aq. acetone, 1:1) [47]). ^1^H NMR (500 MHz, CDCl_3_+CD_3_OD, δ, ppm): 3.38–3.43 (1H, m, H-2″), 3.45–3.51 (2H, m, H-3″,4″), 3.50–3.53 (1H, m, H-5″), 3.78 (1H, ddd, *J* = 11.6, 7.2, 3.2 Hz, H-6″), 3.82 (3H, s, 4′-OCH_3_), 3.93 (1H, dd, *J* = 11.6, 3.6 Hz, H-6″), 4.92 (1H, d, *J* = 7.2 Hz, H-1″), 6.48 (1H, t, *J* = 2.0 Hz, H-4), 6.66 (1H, t, *J* = 1.6 Hz, H-6), 6.78 (1H, t, *J* = 1.6 Hz, H-2), 6.89 (1H, d, *J* = 16.2 Hz, H-7), 6.95 (2H, brd, *J* = 8.8 Hz, H-3′, 5′), 7.03 (1H, d, *J* = 16.2 Hz, H-8), 7.44 (2H, br. d, *J* = 8.8 Hz, H-2′, 6′) (OH protons not observed); ^13^C NMR (125 MHz, CDCl_3_ +CD_3_OD, δ, ppm): 54.61 (4′-OCH_3_), 61.08 (C-6″), 69.62 (C-4″), 72.89 (C-2″), 76.04 (C-5″), 77.18 (C-3″), 100.49 (C-1″), 102.57 (C-4), 105.69 (C-6), 107.02 (C-1′), 113.51 (C3′, 5′), 125.73 (C-2), 127.19 (C-2′,6′), 128.05 (C-7), 129.61 (C-8), 139.38 (C-1), 157.63 (C-5), 158.26 (C-3), 159.84 (C-4′); IR (KBr, ν, cm^−1^): 3380 (OH), 1600, 1592, 1512, 832, 710 (C=C), 1086, 1012 (sugar); UV (MeOH) λ_max_ (lgε): 222 (3.72), 307 (4.84), 321 (4.65); HRMS: calcd for C_21_H_24_O_8_: 404.1471; found {M − H]^+^ *m*/*z*: 404.1468.

Crystallographic data for compound (**11**): The title compound crystallizes in the chiral triclinic space group P1 with one molecule in the unit cell. Crystallographic data for **11**: C_21_H_24_O_8_, *M* 404.40, triclinic, *P1*, *a* 4.7568(5), *b* 6.9943(7), *c* 14.5539(16) Å, α 93.068(4) β 93.138(5) γ 96.171(5) *V* 479.83(9) Å^3^, *Z* 1, *d*_calcd_ 1.400 g·cm^–3^, *μ*(Mo-*K*α) 0.108 mm^–1^, F(000) 214, (θ 2.8–26.1°, completeness 99.5%), T 296(2) K, colorless, (0.80 × 0.15 × 0.04) mm^3^, transmission 0.7158–0.8620, 7478 measured reflections in index range −5≤ h ≤ 5, −8 ≤ k ≤ 8, −17 ≤ l ≤ 17, 3479 independent (*R*_int_ 0.028), 278 parameters, *R*_1_ 0.0391 (for 3082 observed *I* > 2*σ*(*I*)), *wR*_2_ 0.1066 (all data), GOOF 1.03, Flack-parameter-0.2(5), largest diff. peak and hole 0.16 and −0.20 e.A^−3^.

The structure of compound **11** was solved by direct methods using the SHELXT-2014/5 [48] and refined by the full-matrix least-squares method used all F2 in anisotropic approximation using the SHELXL-2018/3 [48]. The hydrogen atoms’ positions were calculated with the riding model, except for OH groups. The hydrogen atom positions for OH groups were located from a difference Fourier map and were refined via isotropic approximation. Absorption correction was applied using the empirical multiscan method with the SADABS program [49]. The title compound crystallizes in the chiral triclinic space group P1 with one molecule in the unit cell. Crystallographic data for the structure have been deposited at the Cambridge Crystallographic Data Centre as supplementary publication no. CCDC 2193450. These data can be obtained free of charge from the Cambridge Crystallographic Data Centre via www.ccdc.cam.ac.uk/data_request/cif (accessed on 9 July 2025). The obtained crystal structures were analyzed for short contacts between non-bonded atoms using PLATON [50] and MERCURY [51] programs.

*Resveratroloside [(E)-resveratrol 4′-O-β-D-glucopyranoside]* (**12**). Colorless crystals. M.p. 243–245 °C (EtOAc), [α]_D_ −61.3 (*c* 0.5, MeOH); (lit.: M.p. 253–255 °C (EtOAc), [α]_D_ −64.5 (*c* 0.7, MeOH) [52]; ^1^H NMR (400 MHz, CDCl_3_ +CD_3_OD, δ, ppm): 3.41–3.59 (4H, m, H-2″,3″,4″, 5″), 3.75 (1H, dd, *J* = 11.6, 5.3 Hz, H-6″b), 3.93 (1H, dd, *J* = 11.6, 2.1 Hz, H-6″a), 4.94 (1H, dd, *J* = 7.6, 7.1 Hz, H-1″), 6.24 (1H, d, *J* = 2.0 Hz, H-4), 6.58, 6.60 (2H, both d, *J* = 2.0 Hz, H-2,6), 6.84 (1H, d, *J* = 16.5 Hz, H(α), 7.01 (1H, d, *J* = 16.5 Hz, H(β)), 7.06 (2H, d, *J* = 8.7 Hz, H-3′,5′), 7.43 (2H, d, *J* = 8.7 Hz, H (2′,6′) (OH protons not observed); ^13^C NMR (101 MHz, CDCl_3_ + CD_3_OD, δ, ppm): 60.72 (C-6″), 69.42 (C-4″), 72.86 (C-2″), 75.85 (C-5″), 78.18 (C-3″), 100.28 (C1″), 101.29 (C-4), 104.23 (C-2,6), 118.01 (C-3′,5′), 126.72 (Cα), 126.79 (C-2′,6′), 127.10 (Cβ), 131.35 (C1′), 139.04 (C1), 156.44 (C4′), 157.46 (C3,5). IR (KBr, ν, cm^−1^): 3346, 3280 (OH), 1601, 1582, 1511, 835, 710 (C=C), 1080, 1010 (sugar); UV λ_max_ (lg ε) EtOH, nm: 212 (4.52), 304 (4.34), 315 (4.25). HRMS: calcd for C_20_H_22_O_8_: 390.1041; found [M − H]^+^ *m*/*z*: 390.1052.

### 3.2. Cell Culture and Cytotoxicity Assay

The HPV-negative human cervical cancer cell line, C33 A (ATCC HTB-31), HPV16-positive human cervical cancer cell line, CaSki (ATCC CRM-CRL-1550), HPV18-positive human cervical cancer cell line, HeLa (ATCC CCL-23), breast cancer (adenocarcinoma MCF-7) (ATCC HTB-22), prostate cancer cell line (DU-145) (ATCC HTB-81) and glioblastoma (T98G, SNB-19) (ATCC CRL-2219) cell lines were obtained from the American Type Culture Collection (ATCC). Normal epithelial VERO cells (Cat. No. T8299) derived from the kidney of an African green monkey were used as the non-cancer control. This cell line was obtained from the cell collection of the State Research Center for Virology and Biotechnology “Vector” of Rospotrebnadzor, Koltsovo, Novosibirsk Region. The cells were cultured in DMEM/F12 medium containing 10% embryonic calf serum, L-glutamine (2 mmol/L), and gentamicin (80 µg/mL) in a CO_2_ incubator at 37 °C. The tested extract and compounds (**1**, **1a** + **3a**, **4**, **6**, **9**, **11**, and **12**) and the reference drug, doxorubicin, were dissolved in DMSO and added to the cellular culture at the required concentrations (the residual concentration of DMSO in the sample did not exceed 1%). Three wells were used for each concentration. The cells that were incubated with only DMSO without the compounds were used as controls. Cells were placed in 96-well microplates and cultivated at 37 °C in 5% CO_2_/95% air for 48 h. Cell viability was assessed through an MTT [3-(4,5-dimethylthiazol-2-yl)-2,5-phenyl-2H-tetrazolium bromide] conversion assay [34], where 1% MTT was added to each well. Four hours later, the medium was removed, leaving the formazan crystals, and isopropanol was added and mixed for 15 min. The optical density of the samples was measured on a Thermo Multiskan FC spectrophotometer (Thermo Fisher Scientific, Waltham, MA, USA) at a wavelength of 570 nm, with a reference of 670 nm. The 50% cytotoxic dose (GI_50_) of each compound (i.e., the compound concentration that lowers the amount of cells to 50% in a culture or decreases the optical density two-fold compared to the control wells) was calculated from the obtained data. All data are presented as the mean ± SEM from at least three independent experiments. The statistical analysis of the results was performed using the Microsoft Excel 2007, STATISTICA 6.0, and GraphPad Prism 5.0 programs and significance was defined as *p* < 0.05 (*t*-test). The results are given as the average value ± standard deviation from the average.

## 4. Conclusions

In conclusion, both phytochemical and biological evaluations of *R. tataricum* growing in Zailiysky Alatau may be rewarding in the discovery of new and potent metabolites. This paper is the first to highlight the chemical composition and cytotoxicity properties of ethanolic extract and its high total phenolic contents. The proposed scheme for the extraction of active components made it possible to easily isolate important secondary metabolites. Twelve phenolic compounds were isolated from the *TBME* (yield 10% from the crude extract or 1.8% from dry rhizomes) and *EtOAc* fractions (yield 30% from the crude extract, or 5.6% from dry rhizomes) of the studied extract. Of interest was the high contents of phenylbutanoid (rhododendron) **1**, hydrolysable tannin β-glucogallin **6**, aryl esters of quinic acid (polyhydroxyphenylpropanoids **7** and **8**) and stilbenes **10**–**12**. The extract and isolated compounds were non-toxic towards normal epithelial VERO cells. Arylbutanoids **1** and **4** exhibited high cytotoxicity towards cervical cancer cell lines CaSki and breast adenocarcinoma MCF-7. Stilbenes **11** and **12** exhibited cytotoxicity towards breast adenocarcinoma cell line MCF-7 and glioblastoma cancer cell line SNB-19 at a low micromolar concentration. The cytotoxicity findings in this study urge for further mechanistic and toxicological evaluations.

## Figures and Tables

**Table 1:** Concentrations of half-maximal inhibition (GI_50_ ± SEM, μM) on five cancer cell lines and VERO cells for compounds **1**, (**1a** + **3a**), **4**, **6**, **9**, **11** and **12**.

Extract or Compound	Growth Inhibition of Cells (GI_50_ ± SEM, μM) ^[a,b]^							
	C33 A (HPV- Negative)	CaSki (HPV-16)	HeLa (HPV-18)	MCF-7	DU-145	SNB-19	T98G	VERO
*TBME* fraction	41 ± 3.6	38 ± 1.2	31 ± 2.2	39 ± 1.6	51 ± 3.1	43 ± 5.4	66 ± 4.9	91 ± 7.8
*EtOAc* fraction	61 ± 3.2	41 ± 2.8	61 ± 6.6	43 ± 2.9	49 ± 3.4	76 ± 6.1	49 ± 3.8	83 ± 4.6
Ethanol extract	55 ± 4.2	52 ± 4.8	65 ± 3.8	36 ± 2.8	68 ± 5.2	52 ± 4.5	78 ± 3.1	86 ± 6.3
**1**	27 ± 1.4	12 ± 0.7	16 ± 0.8	10 ± 1.8	21 ± 2.3	23 ± 4.1	29 ± 2.5	>100
**1a** + **3a** (5:1)	25 ± 1.8	19 ± 0.5	19 ± 1.4	17 ± 0.4	28 ± 0.7	37 ± 1.6	45 ± 4.8	>100
**4**	28 ± 0.5	10 ± 0.6	29 ± 0.7	11 ± 1.4	21 ± 0.8	15 ± 0.9	18 ± 1.1	>100
**6**	27 ± 2.6	18 ± 1.4	29 ± 2.4	31 ± 0.7	67 ± 6.4	26 ± 1.2	21 ± 0.8	>100
**9**	19 ± 2.2	20 ± 1.5	23 ± 3.7	21 ± 2.1	60 ± 1.7	33 ± 1.8	19 ± 0.9	>100
**11**	NT	NT	17 ± 0.9	8 ± 1.8	NT	13 ± 1.1	18 ± 3.5	92 ± 7.9
**12**	NT	NT	18 ± 1.6	7 ± 1.2	NT	9.2 ± 0.8	21 ± 1.7	98 ± 4.8
Doxorubicin	2.5 ± 0.8	10.1 ± 0.7	6.1 ± 0.7	5.2 ± 0.8	15 ± 0.3	7.6 ± 0.7	13 ± 0.5	8 ± 1.8

^[a]^ GI_50_: concentration at which 50% growth inhibition of tumor cells is observed after 48 h incubation. ^[b]^ Experimental results are given as data average values obtained from three independently conducted experiments.

## Data Availability

Data are contained within the article.

## Supplementary Materials

The following supporting information can be downloaded at https://www.mdpi.com/article/10.3390/molecules30142978/s1, (HPLC-analysis of samples of *Rheum tataricum* L.fil. extract (S1–S2); analytical HPLC of compounds **1**, **4**, **9**, **11**, and **12**; NMR (^1^H and ^13^C) spectra of **1**, (**1a** + **3a**), **6**, **9**, **11**, and **12**).
