# Supplementary material for: Isolation and Identification of Secondary Metabolites in Rheum tataricum L.fil. Growing in Kazakhstan and Surveying of Its Anticancer Potential
Source: Molecules. 2025 Jul 15;30(14):2978. doi: 10.3390/molecules30142978 (PMC12299285; doi:10.3390/molecules30142978)
Supplement: Supplementary file 1 [file molecules-30-02978-s001.zip › molecules-3706183-supplementary.pdf]

SUPPLEMENTARY MATERIAL

Title: Isolation and Identification of Secondary Metabolites in *Rheum tataricum* L.fil. Growing in Kazakhstan and Surveying of its Anticancer Potential

Aiman A. Turgunbayeva [1*], Nurgul A. Sultanova [1], Mohammad Saleh Hamad [2], Victor A. Savelyev [3], Elena I. Chernyak [3], Irina Yu. Bagryanskaya [3], Mikhail A. Pokrovsky [3], Andrey G. Pokrovsky [2], Nadezhda G. Gemejiyeva[4] and Elvira E. Shults [3*]

[1] Faculty of Natural Sciences, Department of Chemistry, L.N. Gumilyov Eurasian National University, Satpayeva str., 2, 010008, Astana, Kazakhstan; ayman_88@mail.ru (A.A.T.); nureu@mail.ru (N.A.S.)

[2] Zelman institute for the Medicine and Psychology, Novosibirsk State University, Pirogova Str., 1, 630090 Novosibirsk,Russia; m.khamad@g.nsu.ru (M.S.H.); miha.pokrovsky@gmail.com (M.A.P.); agpok@inbox.ru (A.G.P.)

[3] Novosibirsk Institute of Organic Chemistry, Siberian Branch of the Russian Academy of Sciences, 630090 Novosibirsk, Russia; vicsav@nioch.nsc.ru; chernyak@nioch.nsc.ru (E.I.C); schultz@nioch.nsc.ru (E.E.S.)

[4] Institute of Botany and Phytointroduction FWC of the Ministry of Ecology and natural resources of the Republic of Kazakhstan, Timiryazev Str., 36 D, 050040, Almaty, Kazakhstan (N.G.G.); ngemed58@mail.ru

* Correspondence: (A.A.T.); schultz@nioch.nsc.ru (E.E.S.)

Corresponding authors:

E-mail address: schultz@nioch.nsc.ru (E.E. Shults), ayman_88@mail.ru (A. A. Turgunbayeva)

Table of contents

			Page
1	Figure S1. HPLC-analysis of a sample of *Rheum tataricum* **L.fil.** *TBME* fraction of ethanol extract	S1	3
2	Figure S2. HPLC-analysis of a sample of *Rheum tataricum* **L.fil.** *EtOAc* fraction of ethanol extract	S2	4
3	Figure S3. HPLC-analysis of rhododendrin **1** isolated from *Rheum tataricum* **L.fil.** ethanol extract	S3	5
4	Figure S4. HPLC-analysis of (*R*)-4-(4-Hydroxyphenyl)-2-butanol ((-)-Rhododendrol) **4** isolated from *R. tataricum* L.fil. ethanol extract.	S4	6
5	Figure S5. HPLC-analysis of raspberry ketone **9** isolated from *R. tataricum* L.fil. ethanol extract.	S5	7
6	Figure S6. HPLC-analysis of desoxyrhaponticin **11** isolated from *R. tataricum* L.fil. ethanol extract.	S6	8
7	Figure S7. HPLC-analysis of resveratroloside **12** isolated from *R. tataricum* L.fil. ethanol extract.	S7	9
8	Figure S8. ^{1}H NMR spectrum of acetates (**1a** + **3a**) ($CDCl_3$, 500 MHz)	S8	10
9	Figure S9. ^{13}C NMR spectrum for compound (**1a** + **3a**) ($CDCl_3$, 125 MHz)	S9	11
10	Figure S10. ^{1}H NMR spectrum of rhododendrin **1** $(CD_3)_2SO$, 400 MHz)	S10	12
11	Figure S11. ^{13}C NMR spectrum of rhododendrin **1** $(CD_3)_2SO$, 101 MHz)	S11	13
12	Figure S12. ^{1}H NMR spectrum of β-glucogallin **6** ($CDCl_3$+CD_3OD, 400 MHz)	S12	14
13	Figure S13. ^{13}C NMR spectrum of β-glucogallin **6** ($CDCl_3$+CD_3OD, 101 MHz)	S13	15
14	Figure S14. ^{1}H NMR spectrum of raspberry ketone **9** ($CDCl_3$, 400 MHz	S14	16
15	Figure S15. ^{13}C NMR spectrum of raspberry ketone **9** ($CDCl_3$, 101 MHz	S15	17
16	Figure S16. ^{1}H NMR spectrum of desoxyrhaponticin **11** ($CDCl_3$+CD_3OD, 500 MHz)	S16	18
17	Figure S17. ^{13}C NMR spectrum of desoxyrhaponticin **11** ($CDCl_3$+CD_3OD, 125 MHz)	S17	19
18	Figure S18. ^{1}H NMR spectrum of resveratroloside **12** ($CDCl_3$+CD_3OD, 400 MHz)	S18	20
19	Figure S19. ^{13}C NMR spectrum of resveratroloside **12** ($CDCl_3$+CD_3OD, 101 MHz)	S19	21
20	Figure S20. The packing diagram of compound **11** viewed down the *a*-axis.	S20	22
21	Table S1. Table S1. Parameters of H-bond for compound **11.**	Table S1	22

HPLC analysis was performed on an Agilent LC 1100 chromatograph (Agilent Technologies, Santa Clara, CA, USA) equipped with a quaternary pump, an autosampler, and a diode array detector. The chromatographic conditions were as follows: a ZORBAX SB-C18 column (4.6 × 150 mm, 5 μm) (Agilent Technol.); a mobile phase consisting of methanol/0.1% (v/v) trifluoroacetic acid in an H_2O gradient; the methanol percentage in the gradient was 20–95% (minutes 0–30); the flow rate was 0.6 mL/min; the injection volume was 2 μL; and the detection was performed simultaneously at wavelengths: 254, 280 and 320nm.

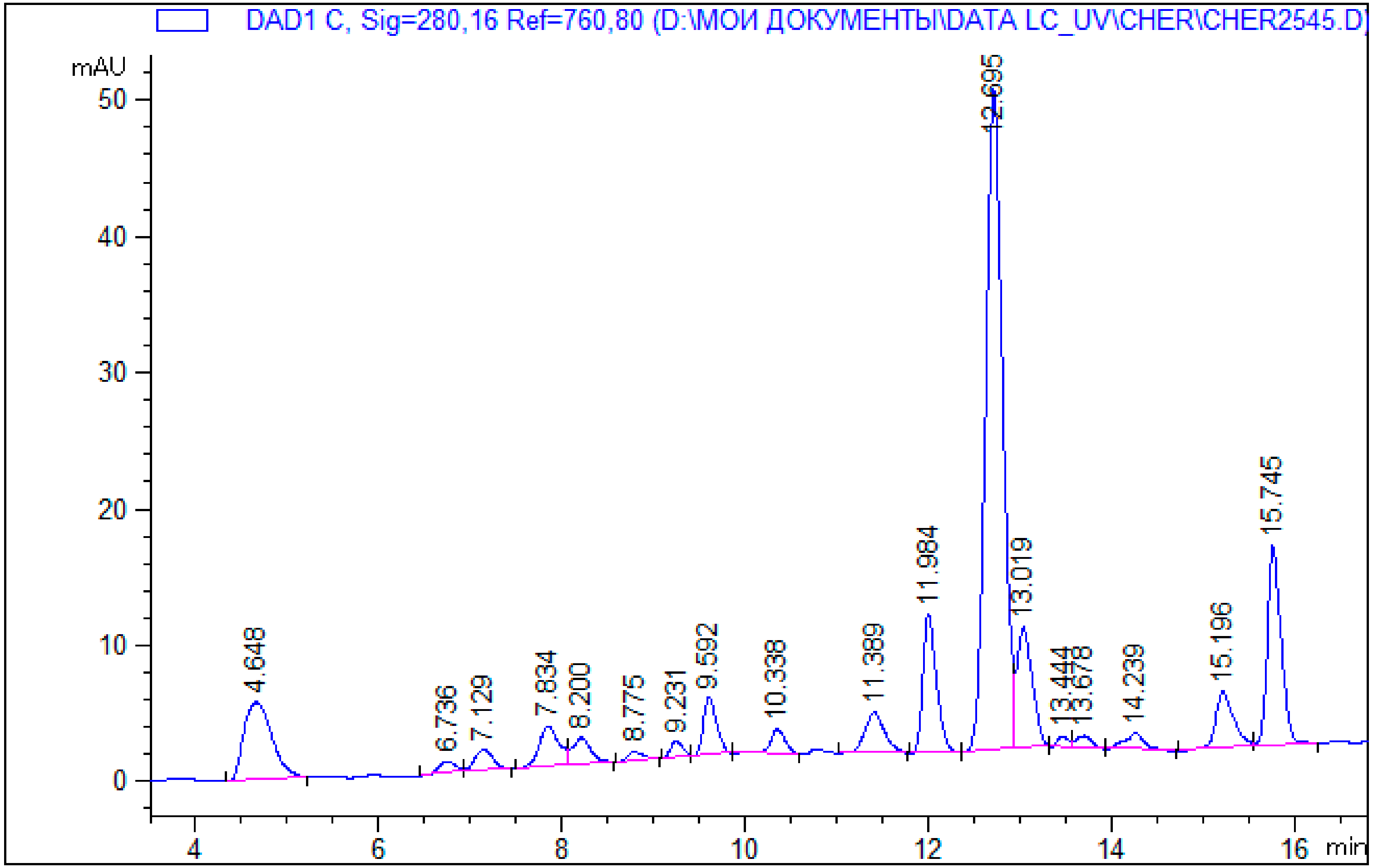

Figure S1. The chromatographic profile of a sample of *TBME* fraction of *Rheum tataricum* L.fil. ethanol extract analyzed by a ZORBAX SB-C18 column.

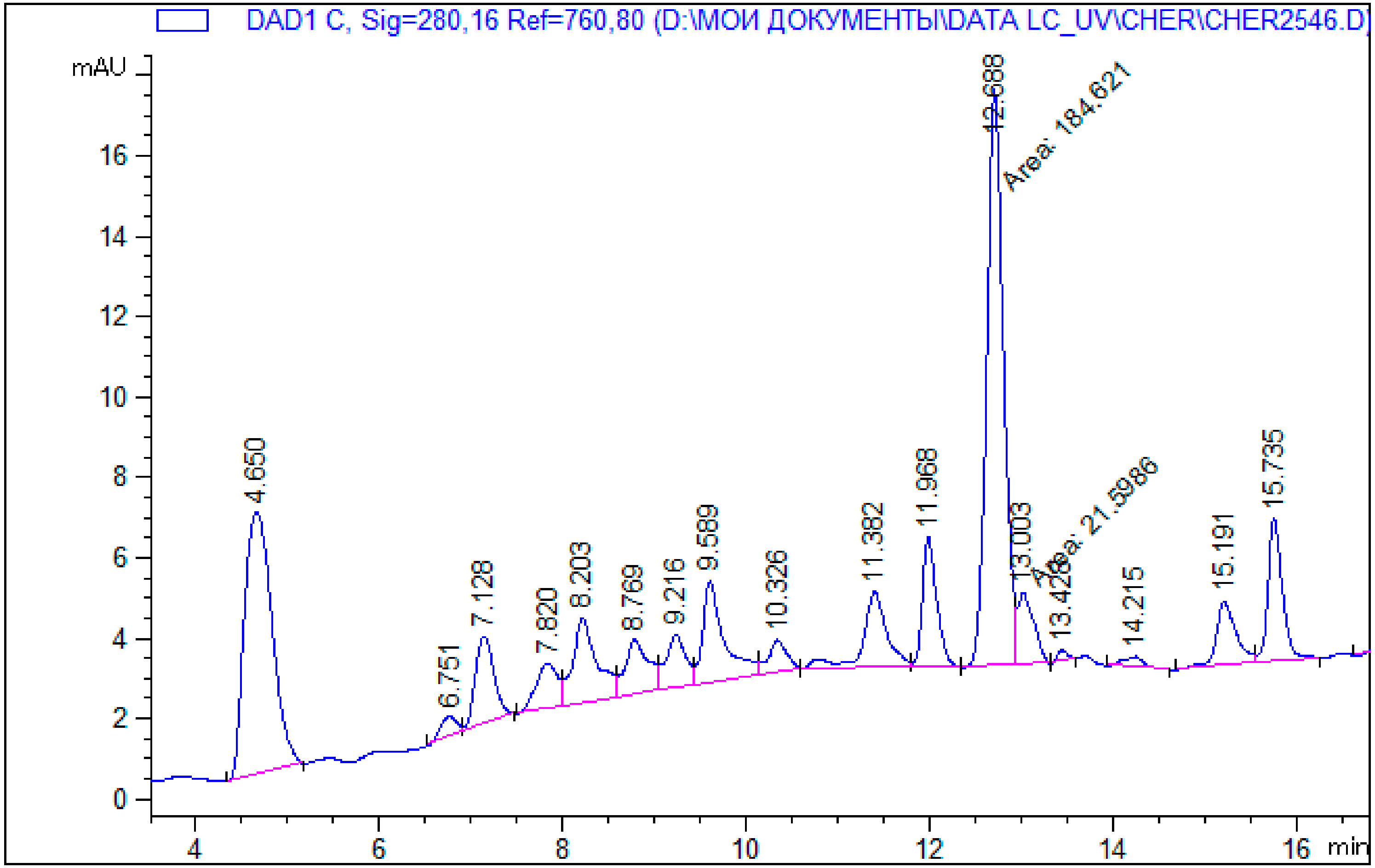

Figure S2. The chromatographic profile of a sample of *EtOAc* fraction of *Rheum tataricum* L.fil. ethanol extract analyzed by a ZORBAX SB-C18 column.

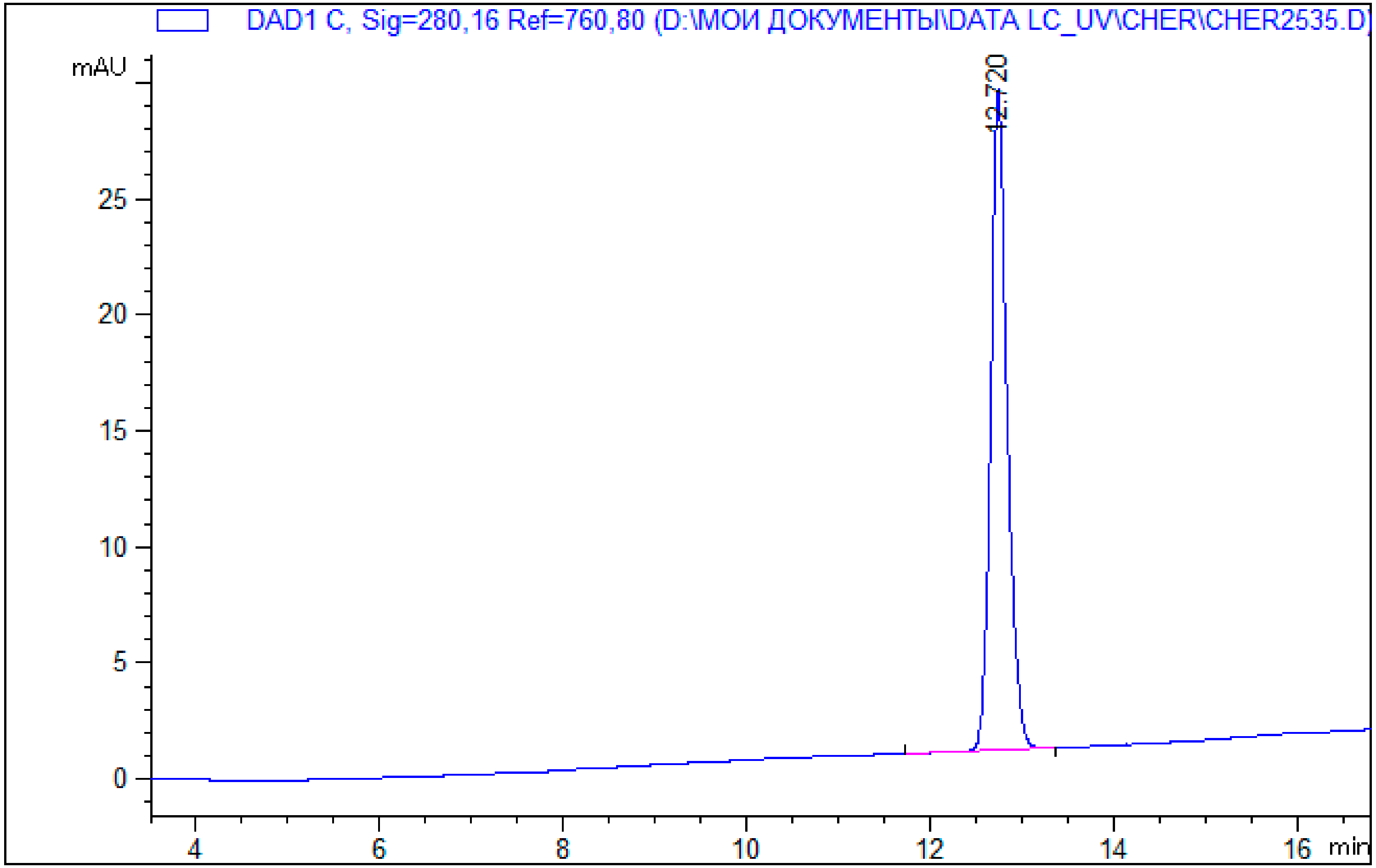

Figure S3. HPLC-analysis of rhododendrin **1** isolated from *Rheum tataricum* L.fil. ethanol extract.

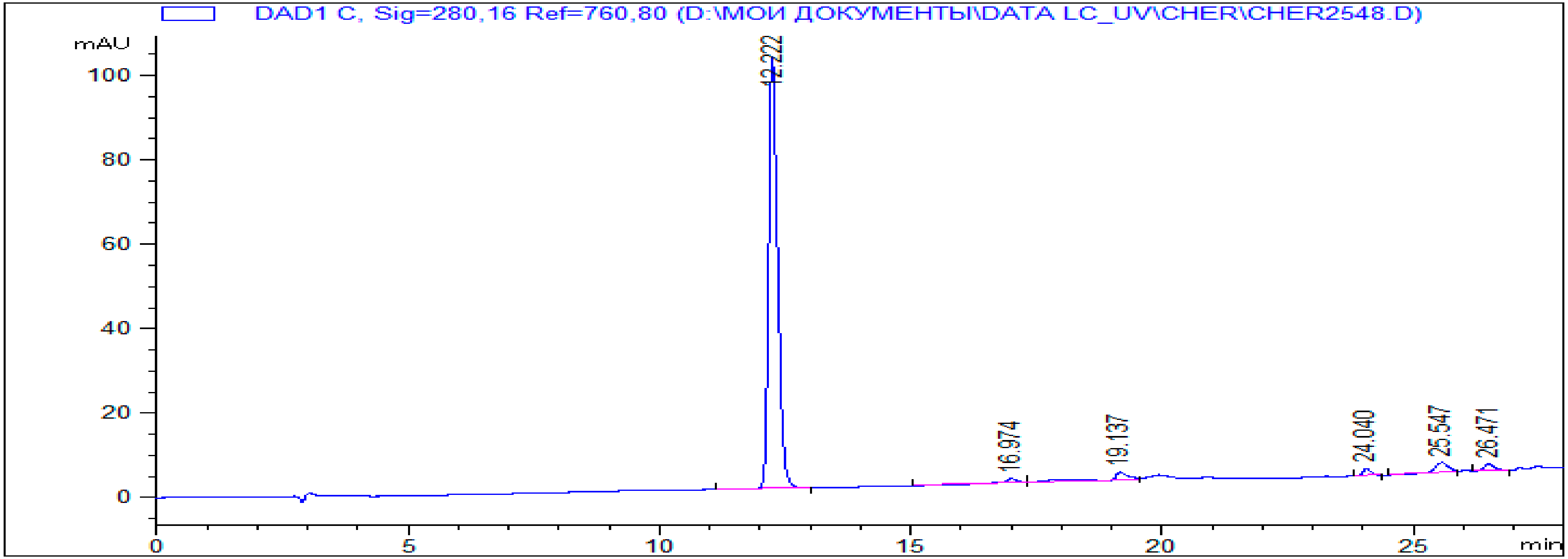

Figure S4. HPLC-analysis of (*R*)-4-(4-Hydroxyphenyl)-2-butanol ((-)-Rhododendrol) **4** isolated from *Rheum tataricum* L.fil. ethanol extract.

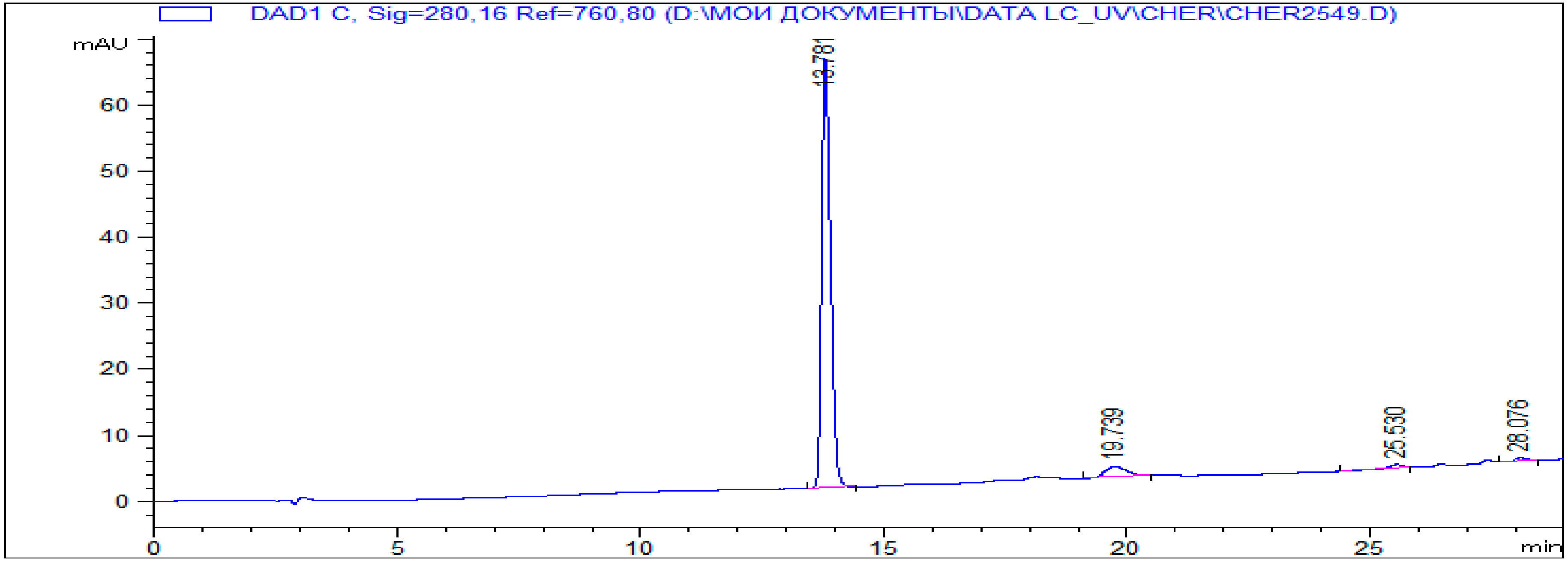

Figure S5. HPLC-analysis of raspberry ketone **9** isolated from *R. tataricum* L.fil. ethanol extract.

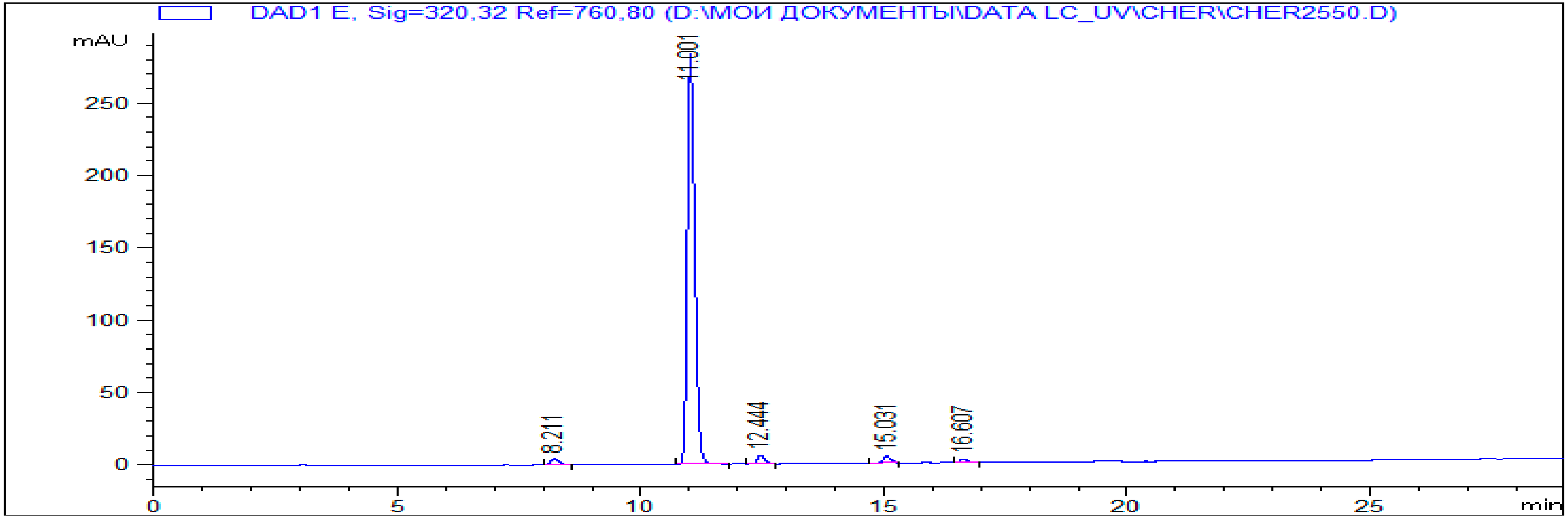

Figure S6. HPLC-analysis of desoxyrhaponticin **11** isolated from *R. tataricum* L.fil. ethanol extract.

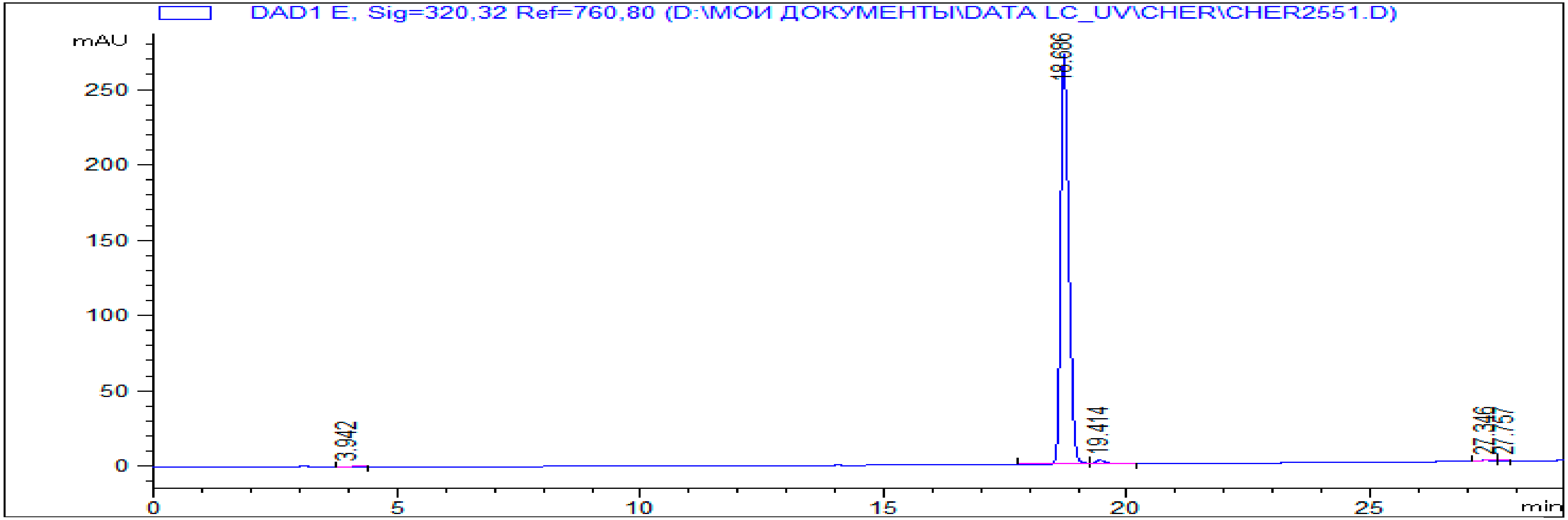

Figure S7. HPLC-analysis of resveratroloside **12** isolated from *R. tataricum* L.fil. ethanol extract.

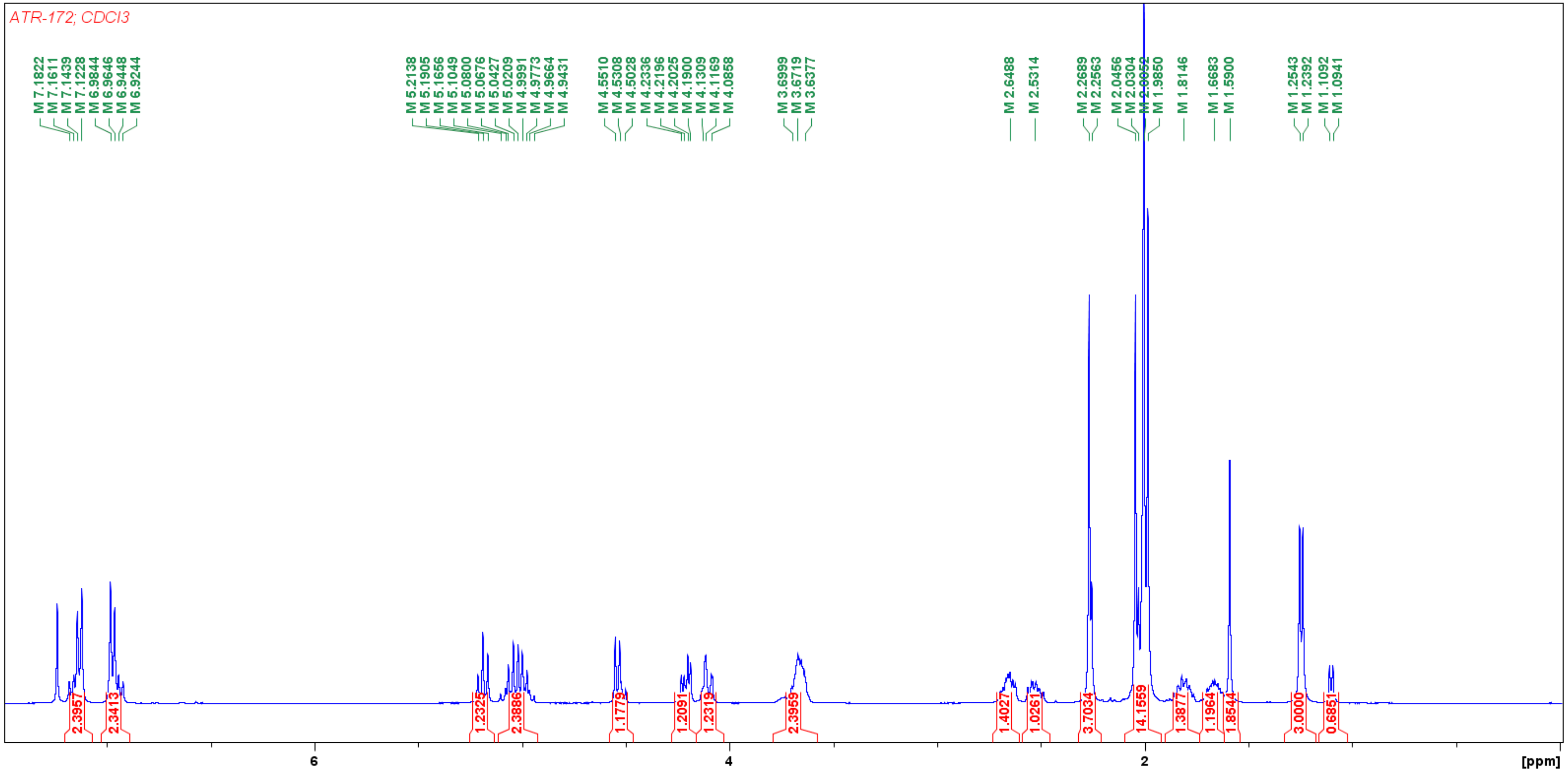

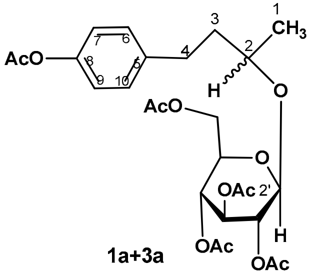

1a+3a

Figure S8. 1H NMR spectrum of acetates (**1a** + **3a**) ($CDCl_3$, 500 MHz)

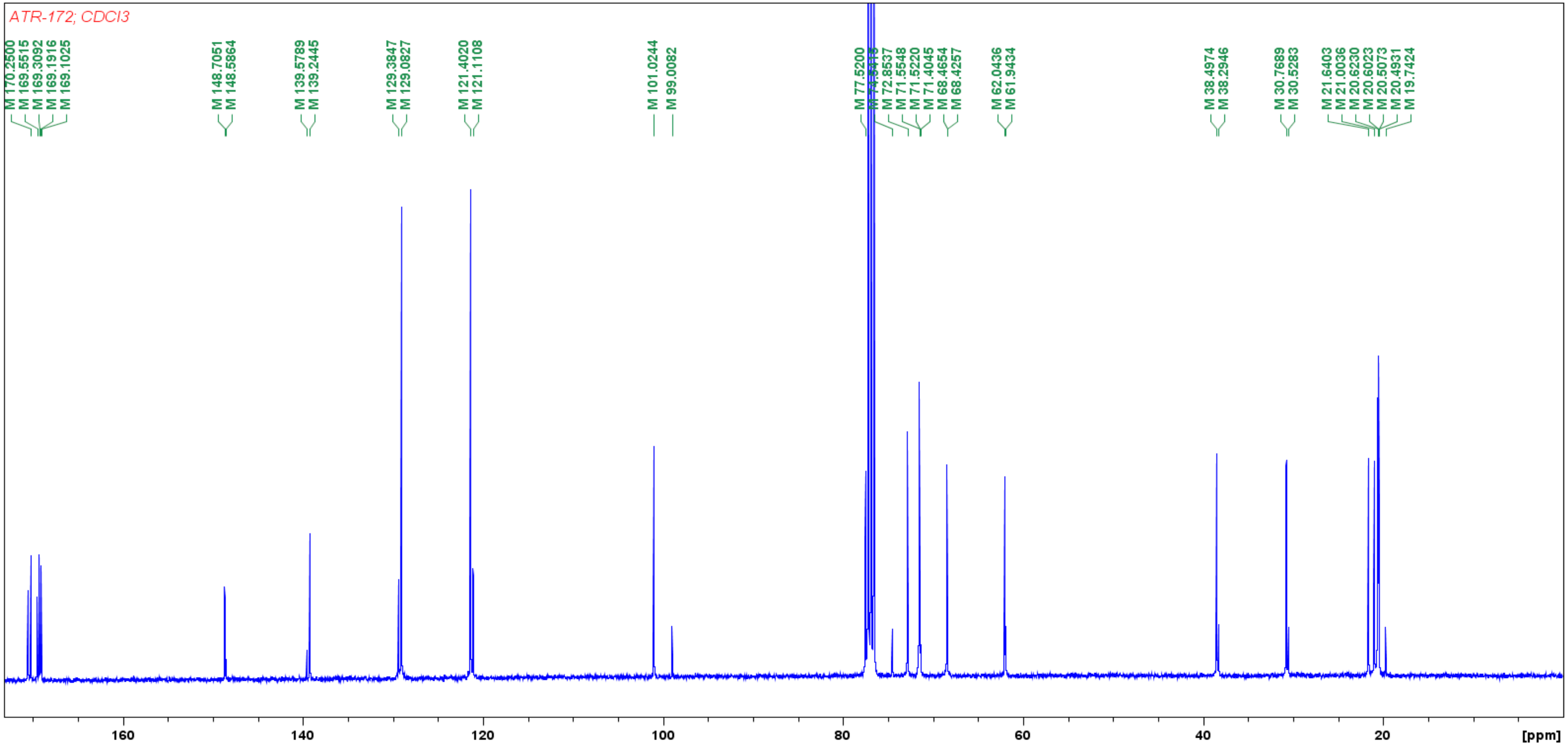

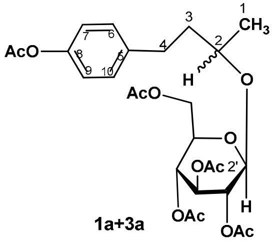

1a+3a

Figure S9. 13C NMR spectrum of acetates (**1a** + **3a**) ($CDCl_3$, 125 MHz)

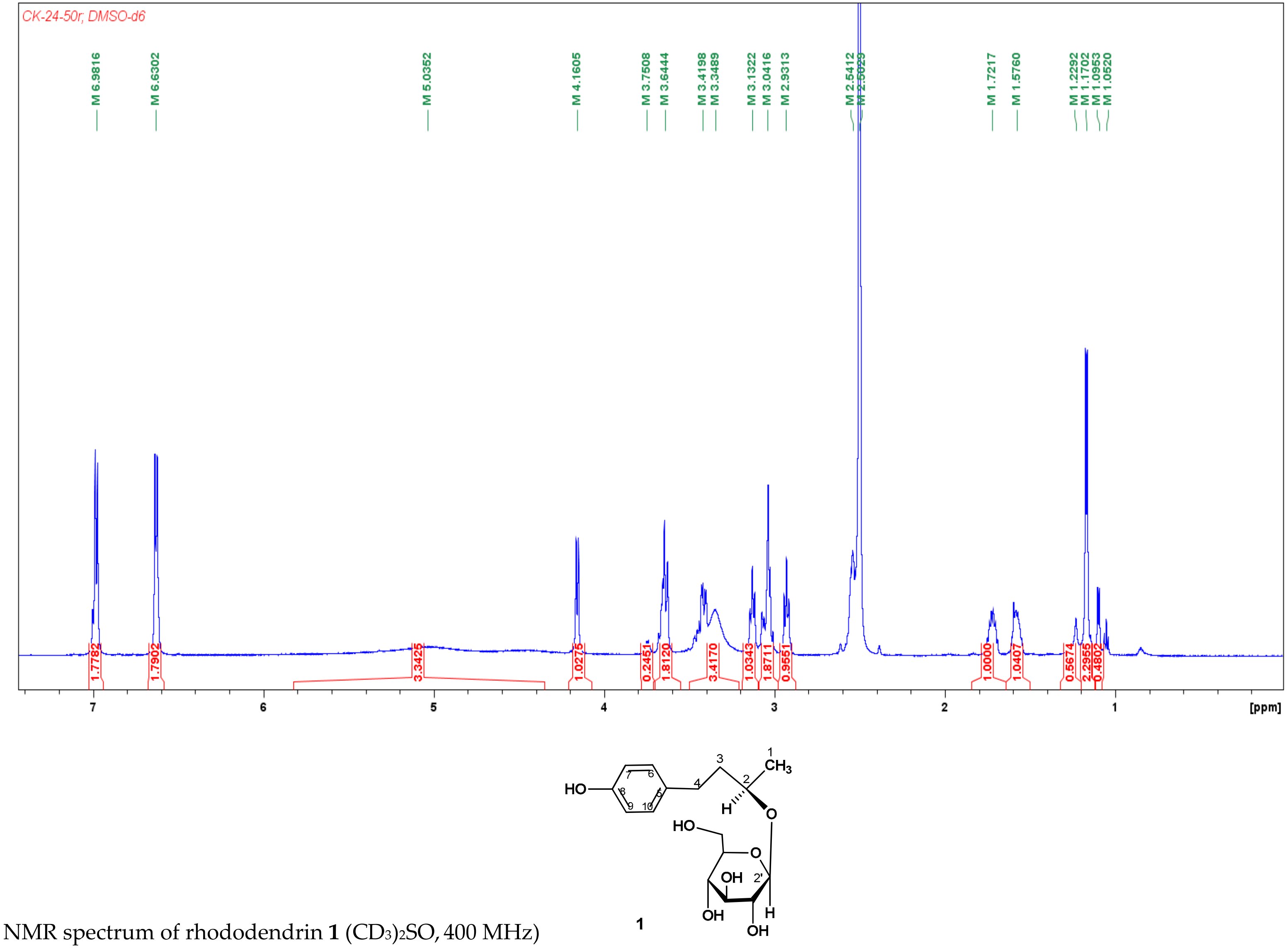

Figure S10. 1H NMR spectrum of rhododendrin **1** $(CD_3)_2SO$, 400 MHz)

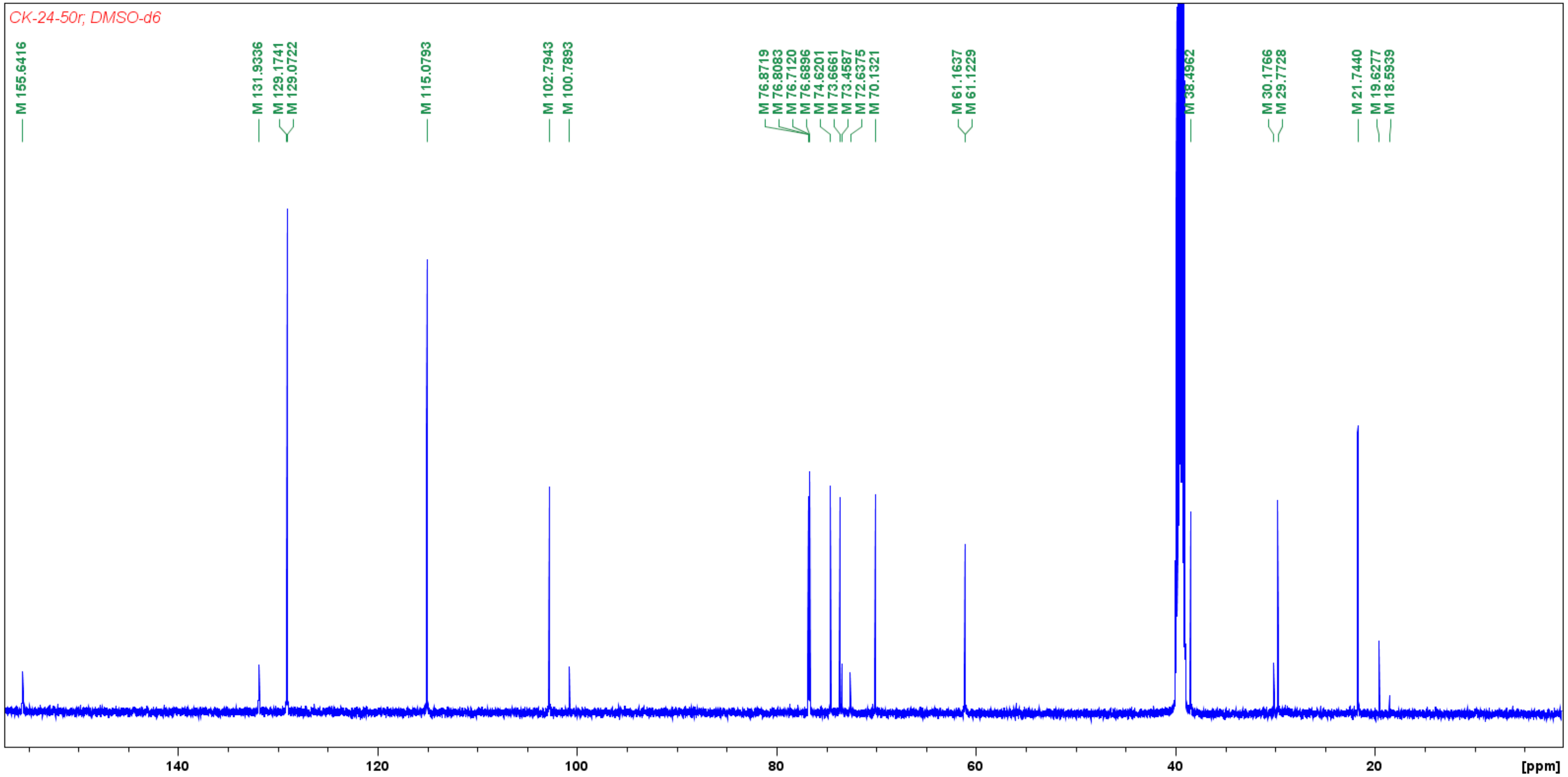

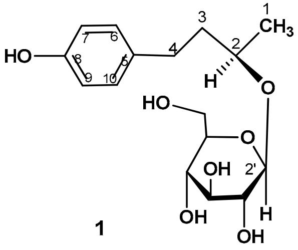

1

Figure S11. ^{13}C NMR spectrum of rhododendrin **1** ($(CD_3)_2SO$, 101 MHz)

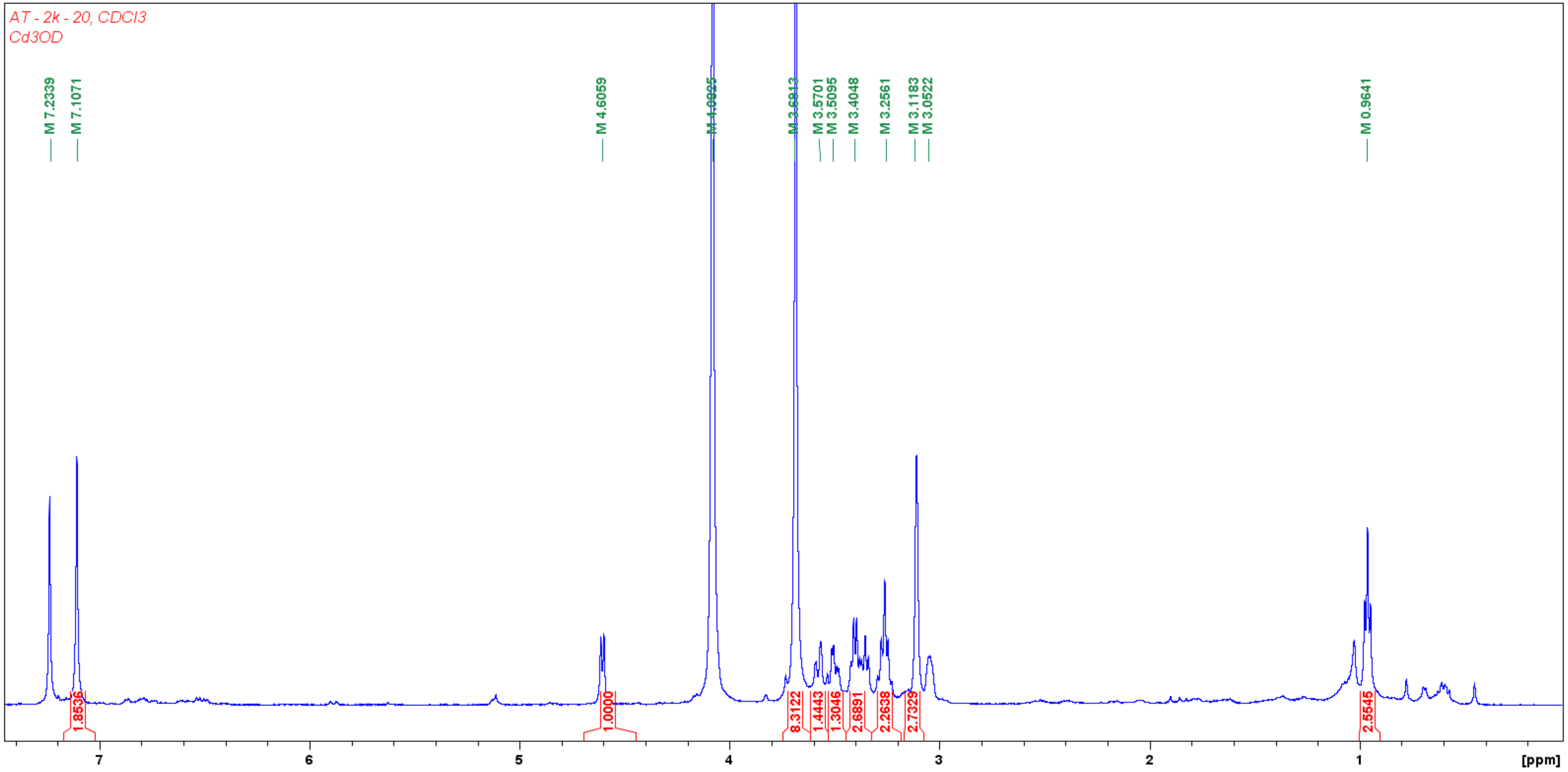

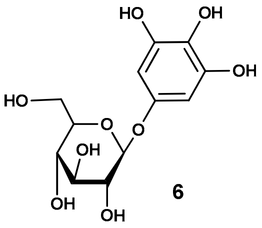

6

Figure S12. ^{1}H NMR spectrum of β-glucogallin **6** ($CDCl_3$+CD_3OD, 400 MHz)

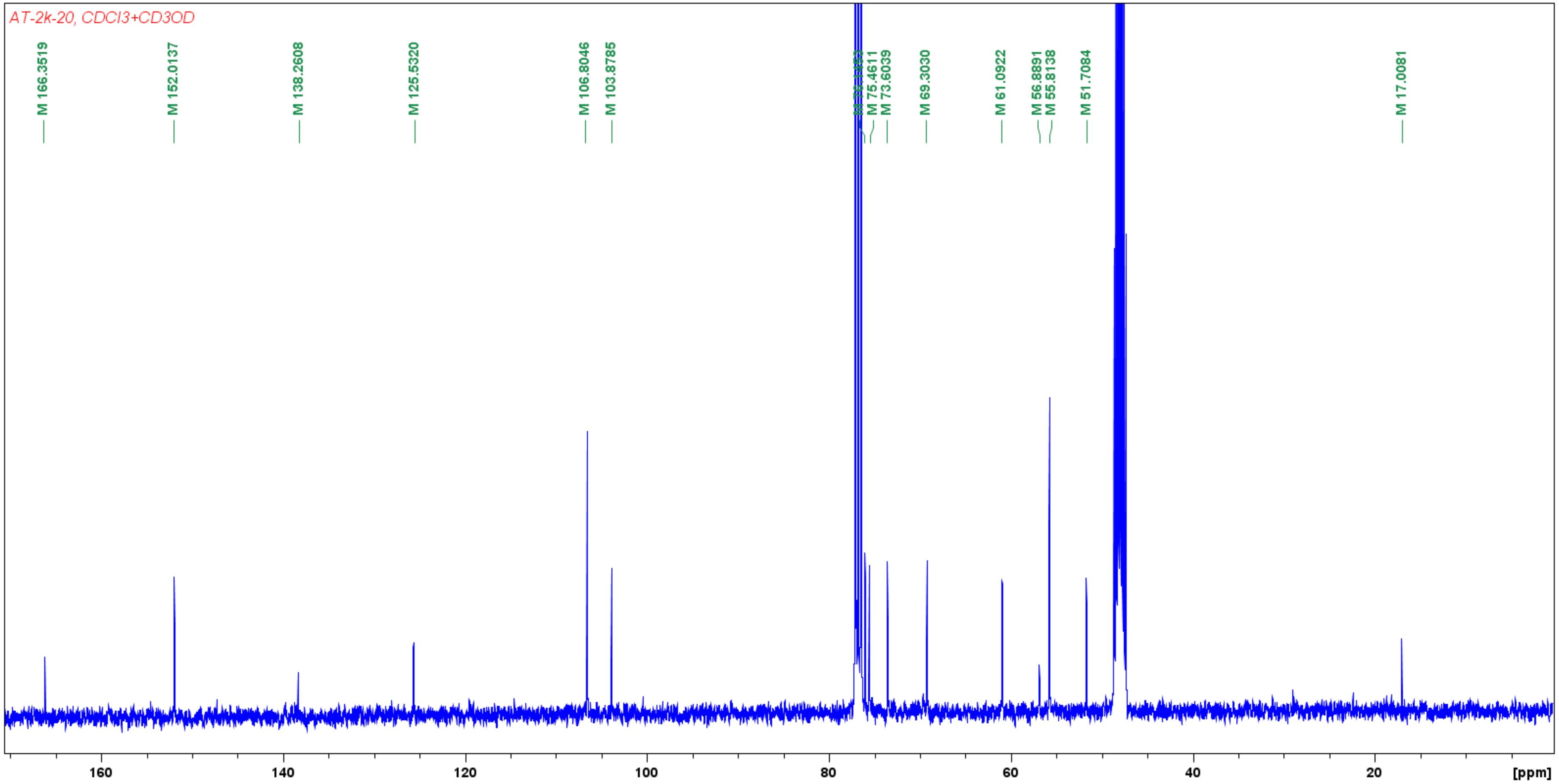

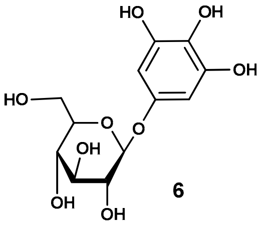

6

Figure S13. ^{13}C NMR spectrum of β-glucogallin **6** ($CDCl_3$+CD_3OD, 101 MHz)

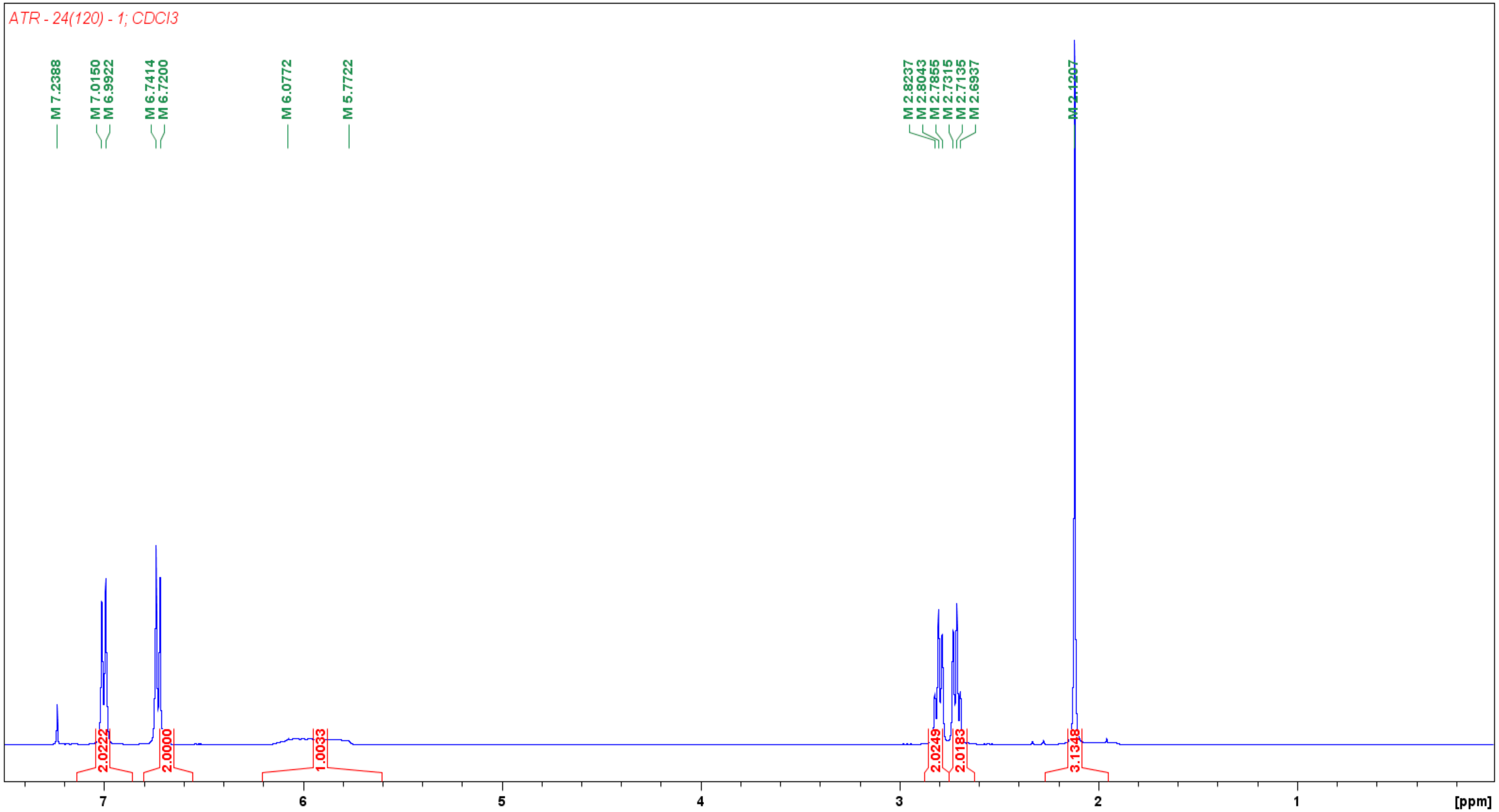

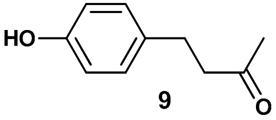

9

Figure S14. ^{1}H NMR spectrum of raspberry ketone **9** ($CDCl_3$, 400 MHz)

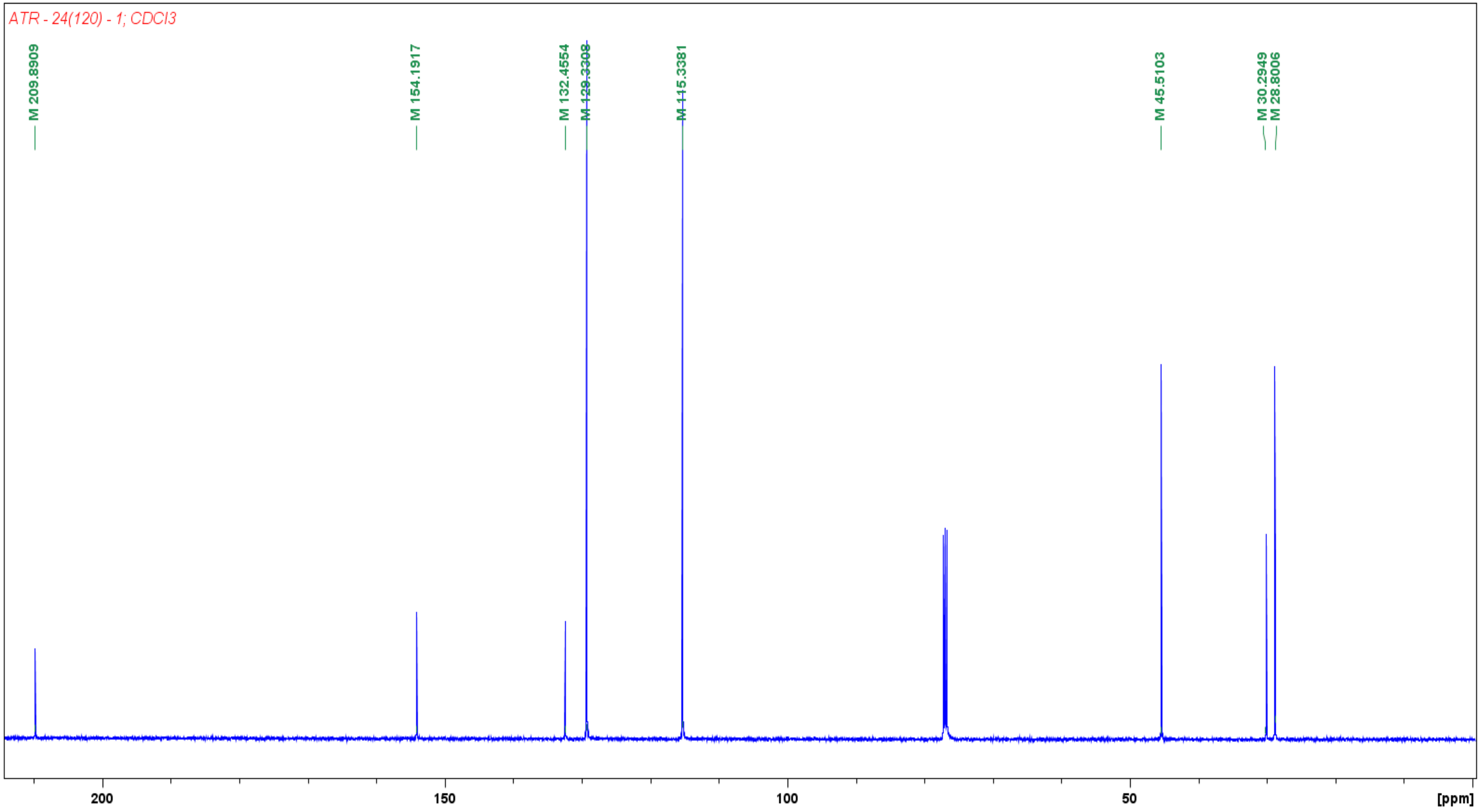

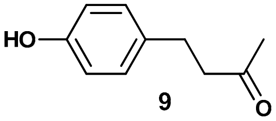

9

Figure S15. ^{13}C NMR spectrum of raspberry ketone **9** ($CDCl_3$, 101 MHz)

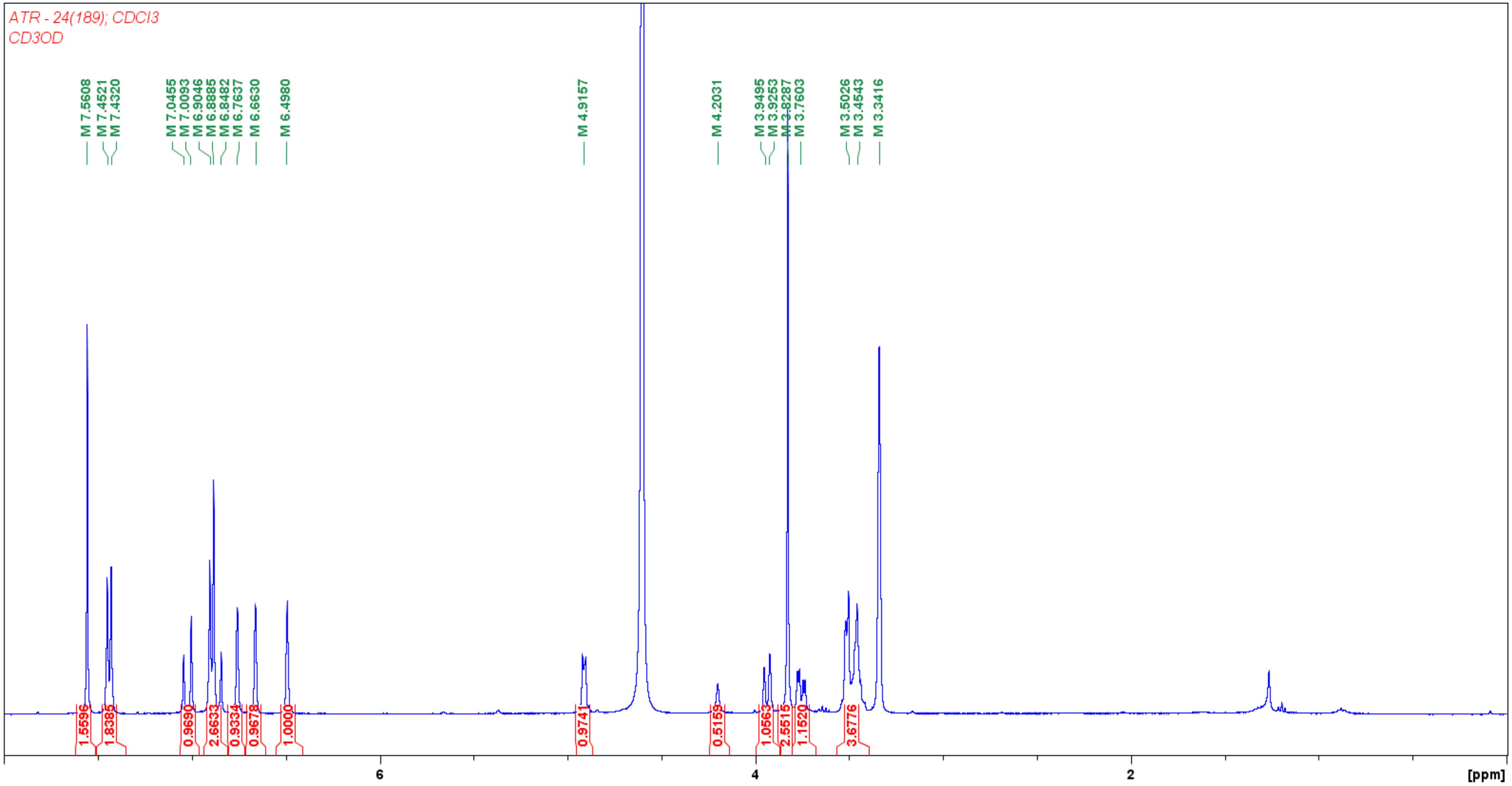

Figure S16. ^{1}H NMR spectrum of desoxyrhaponticin **11** ($CDCl_3$+CD_3OD, 500 MHz)

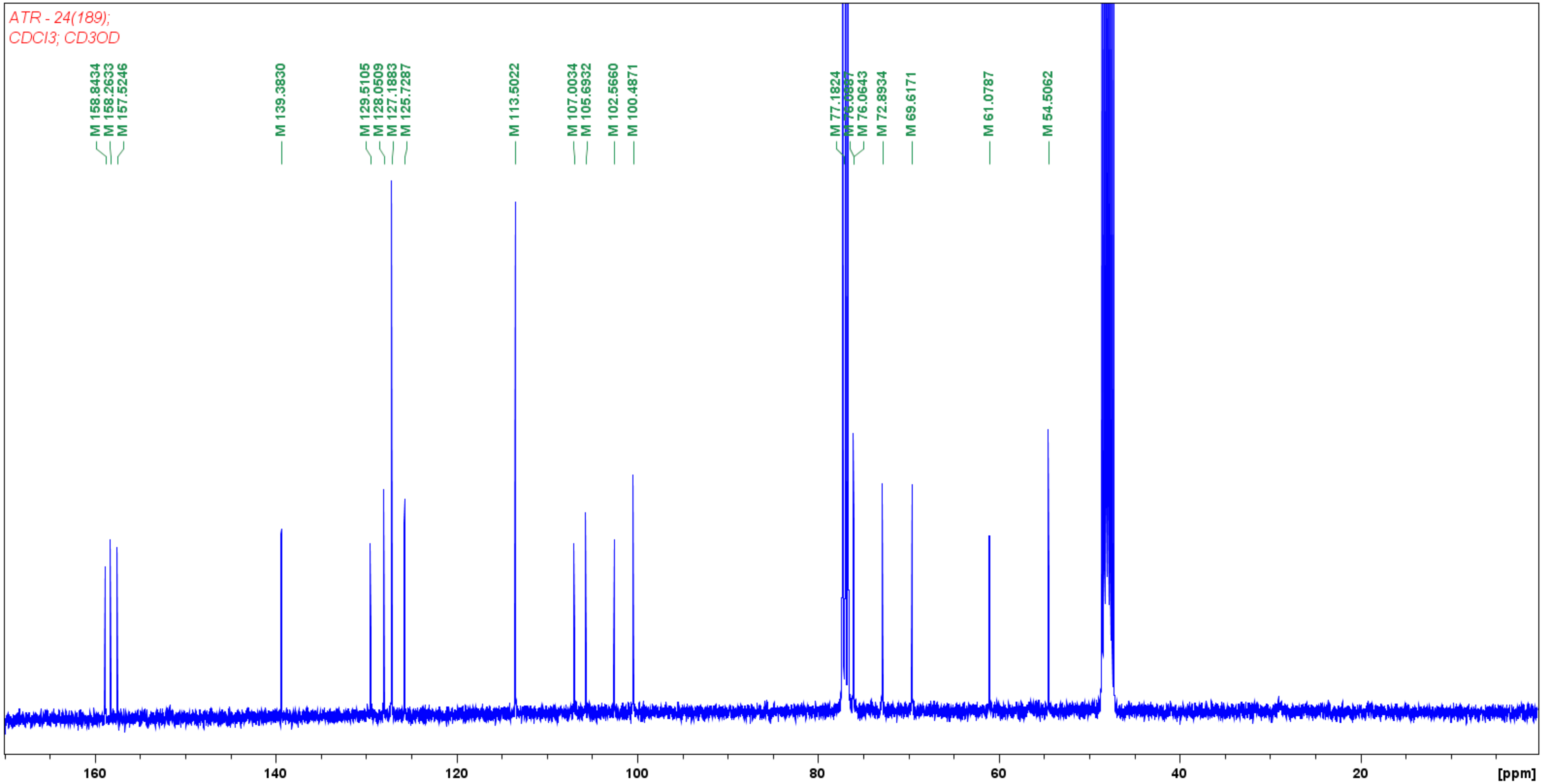

Figure S17. ^{13}C NMR spectrum of desoxyrhaponticin **11** ($CDCl_3$+CD_3OD, 125 MHz)

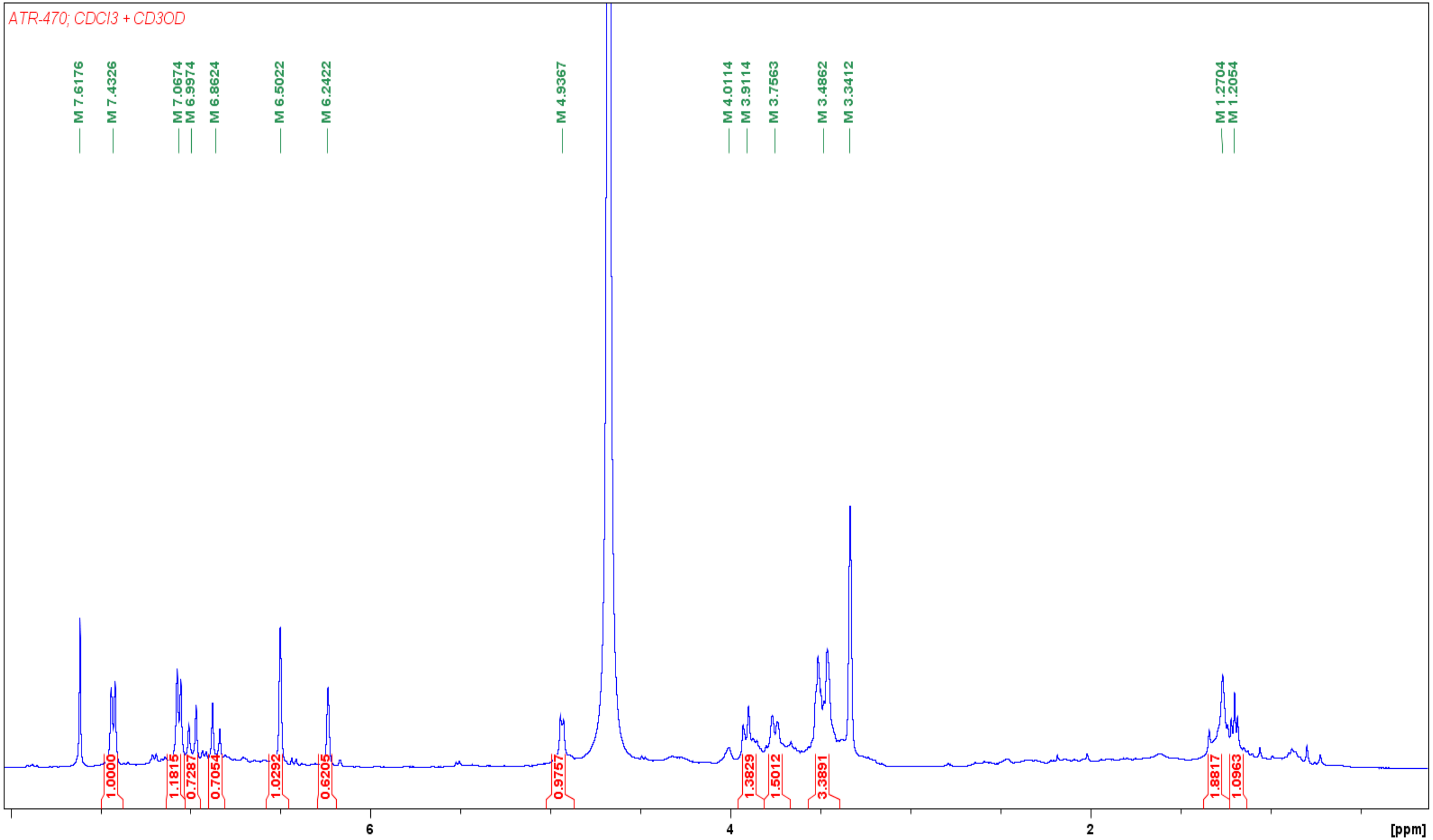

Figure S18. ^{1}H NMR spectrum of resveratroloside **12** ($CDCl_3$+CD_3OD, 400 MHz).

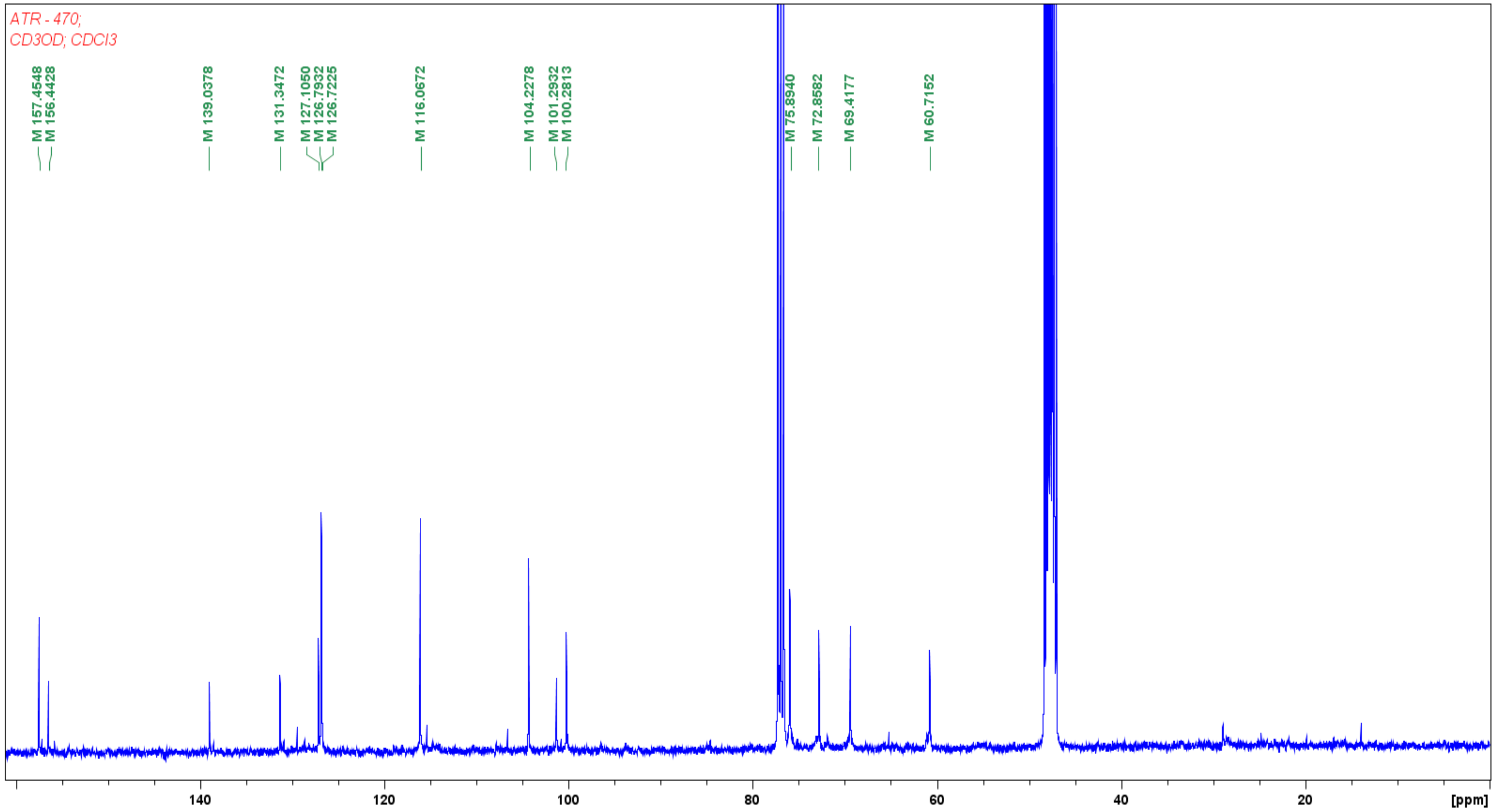

Figure S19. ^{13}C NMR spectrum of resveratroloside **12** ($CDCl_3$+CD_3OD, 101 MHz).

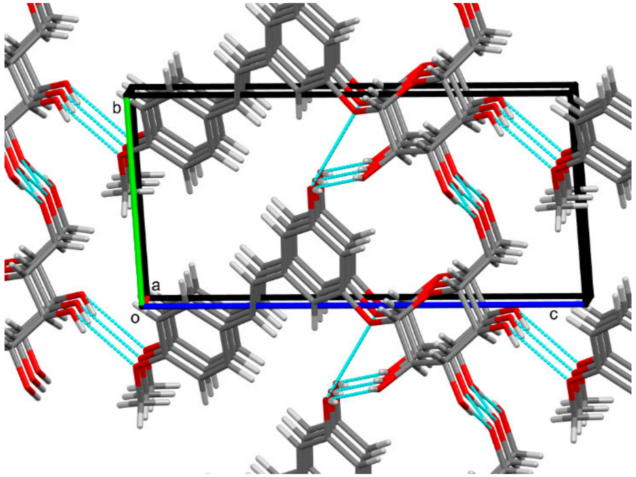

Figure S20. The packing diagram of compound **11** viewed down the *a*-axis.

Table S1. Parameters of H-bond for compound **11.**

H-bond	D-H, Å	H…A, Å	D…A, Å	D-H…A, °
O2-H…O8	0.85(7)	2.06(7)	2.838(4)	15296)
O5-H…O7	0.76(5)	2.00(5)	2.767(3)	176(6)
**O6-H…O3**	0.74(5)	2.13(5)	2.808(4)	153(6)
O7-H…O5	0.95(5)	1.77(5)	2.714(3)	176(5)
O8-H…O2	0.96(5)	2.38(5)	3.205(4)	144(4)
